# Prediction of Water Quality Parameters in the Paraopeba River Basin Using Remote Sensing Products and Machine Learning

**DOI:** 10.3390/s26010018

**Published:** 2025-12-19

**Authors:** Rafael Luís Silva Dias, Ricardo Santos Silva Amorim, Demetrius David da Silva, Elpídio Inácio Fernandes-Filho, Gustavo Vieira Veloso, Ronam Henrique Fonseca Macedo

**Affiliations:** 1 Department of Agricultural Engineering, Universidade Federal de Viçosa, Viçosa 36570-900, MG, Brazil; 2 Department of Soil and Plant Nutrition, Universidade Federal de Viçosa, Viçosa 36570-900, MG, Brazil; 3 Department of Civil Engineering, Universidade Federal de Viçosa, Viçosa 36570-900, MG, Brazil

**Keywords:** freshwater bodies, extreme events, PlanetScope, lentic and lotic environments

## Abstract

Monitoring surface water quality is essential for assessing water resources and identifying their quality patterns. Traditional monitoring methods, based on conventional point-sampling stations, are reliable but costly and limited in frequency and spatial coverage. These constraints hinder the ability to evaluate water quality parameters at the temporal and spatial scales required to detect the effects of extreme events on aquatic systems. Satellite imagery offers a viable complementary alternative to enhance the temporal and spatial monitoring scales of traditional assessment methods. However, limitations related to spectral, spatial, temporal, and/or radiometric resolution still pose significant challenges to prediction accuracy. This study aimed to propose a methodology for predicting optically active and inactive water quality parameters in lotic and lentic environments using remote-sensing data and machine-learning techniques. Three remote-sensing datasets were organized and evaluated: (i) data extracted from Sentinel-2 imagery; (ii) data obtained from raw PlanetScope (PS) imagery; and (iii) data from PS imagery normalized using the methodology developed by Dias. Data on water quality parameters were collected from 24 monitoring stations located along the Paraopeba River channel and the Três Marias Reservoir, covering the period from 2016 to 2023. Four machine-learning algorithms were applied to predict water quality parameters: Random Forest, k-Nearest Neighbors, Support Vector Machines with Radial Basis Function Kernel, and Cubist. Model performance was evaluated using four statistical metrics: root-mean-square error, mean absolute error, Lin′s concordance correlation coefficient, and the coefficient of determination. Models based on normalized PS data achieved the best performance in parameter estimation. Additionally, decision-tree-based algorithms showed superior generalization capability, outperforming the other models tested. The proposed methodology proved suitable for this type of analysis, confirming not only the applicability of PS data but also providing relevant insights for its use in diverse environmental-monitoring applications.

## 1. Introduction

Continental surface water bodies, such as rivers, lakes, reservoirs, and streams, are essential sources of potable water and support multiple uses, including recreation, industry, agriculture, energy generation, transportation, and fishing [1,2]. They also play a fundamental role in maintaining biodiversity and regulating hydrological flow [3]. Therefore, continuous monitoring of water quantity and quality is crucial for understanding natural and anthropogenic processes that influence aquatic systems and for supporting conservation actions [4].

Conventional monitoring of water quality, based on discrete point sampling, presents important limitations, especially during extreme events. Low sampling frequency and the limited spatial distribution of monitoring stations hinder the detection of spatiotemporal variability in water quality, particularly along lake and reservoir margins or following intense rainfall [5,6]. In such situations, continuous and spatially detailed monitoring becomes essential to complement traditional approaches and to more accurately assess the impacts of events such as dam failures and heavy precipitation.

Remote sensing techniques have emerged as effective alternatives for the continuous monitoring of optically active water quality parameters because of their broad spatial coverage, adequate temporal resolution, and favorable cost–benefit ratio. These techniques have been widely applied to estimate chlorophyll-a (Chla), turbidity (T), colored dissolved organic matter (CDOM), total suspended solids (TSS), and Secchi disk depth (SDD) [7,8,9,10].

In contrast, relatively few studies have focused on predicting non-optically active parameters such as phosphorus (P), nitrogen (N), chemical oxygen demand (COD), dis-solved oxygen (DO), and iron (Fe) [11,12]. These parameters exhibit weak or no relation-ships with spectral bands, which limits their direct detection by orbital sensors [13,14,15]. Consequently, their prediction relies on indirect approaches that explore correlations with optically active parameters, environmental variables, and machine-learning algorithms [12,16,17].

Machine-learning algorithms have become increasingly prominent in water-quality modeling due to their ability to represent highly nonlinear relationships among environmental variables [18]. These methods have been applied across a variety of monitoring contexts, often outperforming traditional approaches for several water-quality parameters [19,20]. Recent studies, for example, demonstrate that backpropagation neural networks can accurately estimate indicators such as chemical oxygen demand (COD), permanganate index, total nitrogen (TN), and total phosphorus (TP) [21,22]. In addition to neural networks, techniques such as decision trees, support vector machines, random forests, and other supervised learning algorithms have shown broad applicability in aquatic systems [12,17,23,24].

These methodological limitations are compounded by constraints inherent to current orbital platforms such as Landsat, MODIS, and Sentinel-2 (S2), which have moderate-to-low spatial resolutions ranging from 10 to 1000 m. Furthermore, the temporal resolutions of Landsat and S2A/2B satellites are considered moderate—16 and 10 days, respectively [25,26]. As a result, their application for remote detection of water quality in small reservoirs, embayments of large reservoirs, and narrow rivers—where higher-frequency imagery is needed—is limited [27,28].

To overcome such limitations, Planet has deployed a large number of nanosatellites known as Doves. These Doves form a CubeSat 3U (10 × 10 × 30 cm) constellation capable of daily revisit to the same target on Earth′s surface. Since 2016, these satellites have provided imagery with 3.7 m spatial resolution and four spectral bands (B1—Blue: 420–530 nm; B2—Green: 500–590 nm; B3—Red: 610–700 nm; and B4—near-infrared [NIR]: 780–860 nm) [29]. They weigh approximately 4 kg, enabling much faster production and launch compared with traditional satellites. They also do not require a dedicated launch vehicle, as they can be delivered to orbit as secondary payloads [30].

Recognizing these methodological gaps in the literature, this study offers an innovative contribution by integrating the prediction of optically active and inactive water-quality parameters in both lotic and lentic environments, an aspect that remains underexplored, particularly in highly variable contexts such as post-disaster conditions. This approach is strengthened by the inclusion of systematic comparisons among different orbital products, including Sentinel-2, PlanetScope, and radiometrically normalized PlanetScope, which enables the assessment of model predictive performance and the relevance of the specific characteristics of each orbital dataset. Accordingly, the objective of this study is to propose a methodology for predicting optically active and inactive water-quality parameters in lotic and lentic environments using remote-sensing data and machine-learning techniques.

## 2. Materials and Methods

### 2.1. Study Setting

**Figure 1:**
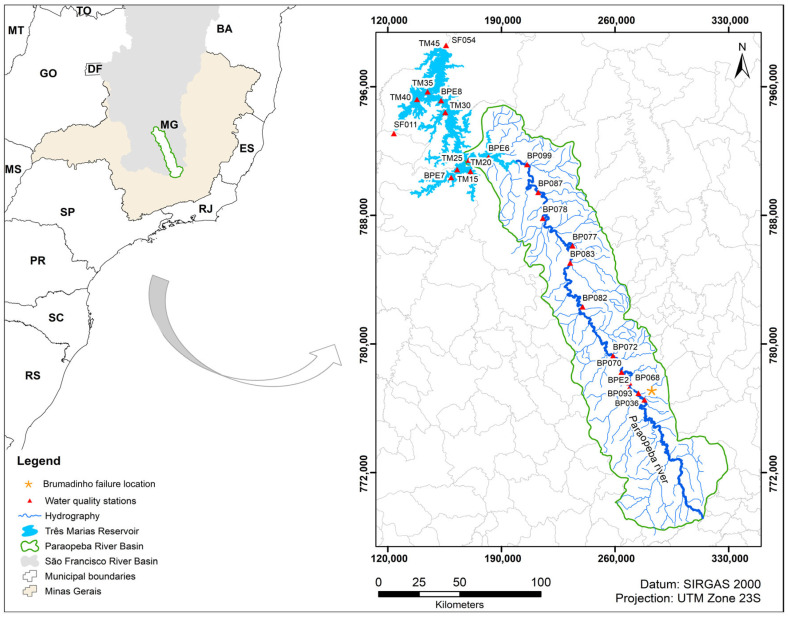
Location of the 24 water-quality monitoring stations distributed along the Paraopeba River Basin and the Três Marias Reservoir in the state of Minas Gerais, Brazil.

To apply and evaluate the proposed methodology, we selected an area located in the central region of the state of Minas Gerais, Brazil (Figure 1), encompassing the Paraopeba River Basin and the Três Marias Reservoir. According to IBGE [31], the Paraopeba River is one of the main tributaries of the São Francisco River, extending 510 km and supplying the Três Marias Reservoir after flowing through 48 municipalities in Minas Gerais.

The Paraopeba River Basin has an average altitude of 720 m and is characterized by predominantly strongly undulating to mountainous terrain [32]. The predominant soil classes in the basin are Red Latosols, Red-Yellow Latosols, Haplic Cambisols, and Humic Cambisols. According to Alvares et al. [33], the climate in the region is classified as tropical Aw, with two well-defined seasons: a rainy summer from October to March and a dry winter from April to September.

This basin was chosen due to its considerable diversity of soils, topography, vegetation cover, and the presence of continental water bodies. Additionally, on 25 January 2019, the Paraopeba River Basin was impacted by one of the most severe socio-environmental disasters in Brazil: the failure of the tailings dam (B-I) at the Córrego do Feijão mine, located in the city of Brumadinho. According to the Government of Minas Gerais [34], approximately 12 million m^3^ of tailings were released, of which an estimated 2 million m^3^ remained in the former B-I area; 7.8 million m^3^ were deposited along the Ferro-Carvão stream channel until its confluence with the Paraopeba River; and the remaining 2.2 million m^3^ reached the main Paraopeba channel.

### 2.2. Hydrological Data

For this study, we used water-quality parameter time-series data (2016–2023) from 24 monitoring stations distributed along the Paraopeba River channel and the Três Marias Reservoir, including station SF054, located downstream of the dam failure (Figure 1 and Table 1). These data included turbidity, TSS, Chla, P, N, COD, DO, and Fe.

Data were obtained from the institutional repository of the Instituto Mineiro de Gestão das Águas (IGAM) (http://repositorioigam.meioambiente.mg.gov.br (accessed on 15 December 2025)), the governmental agency responsible for water-resource monitoring in Minas Gerais. In the basic network, sampling campaigns were conducted quarterly until December 2018. However, after the dam collapse in January 2019, sampling at stations located along the Paraopeba River became monthly. In addition, monitoring records from stations located in the Três Marias Reservoir, under the responsibility of Companhia Energética de Minas Gerais (Cemig), were incorporated into the database.

### 2.3. Remote Sensing Data Acquisition and Processing

To predict water quality parameters, we selected images acquired by the multispectral instrument (MSI) onboard the S2A and S2B satellites as well as PlanetScope (PS) imagery from the three generations of Dove nanosatellites Dove Classic, Dove-R, and Super Dove. Additionally, PS imagery was normalized according to the methodology proposed by Dias [36]. The following subsections describe the characteristics of each product and the processing applied.

#### 2.3.1. Multispectral Instrument/Sentinel-2

In this study, 10 spectral bands from S2 MSI were used, mounted on the S2A and S2B platforms. According to Müller-Wilm [37], the multispectral bands are distributed across different electromagnetic ranges: three visible bands (B2—Blue [490 nm], B3—Green [560 nm], and B4—Red [665 nm]); one NIR band (B8—NIR [842 nm]); four red-edge bands (B5 [705 nm], B6 [740 nm], B7 [783 nm], and B8a [865 nm]); and two shortwave-infrared bands (B11–SWIR [1610 nm] and B12 [2190 nm]). The visible and NIR bands have 10 m spatial resolution, whereas the red-edge and shortwave-infrared bands have 20 m resolution.

S2 scenes were obtained from the Copernicus Open Access Hub (https://dataspace.copernicus.eu, accessed on 15 March 2024). We selected Level-1C top-of-atmosphere reflectance products with no cloud cover and with a maximum temporal difference of two days relative to the in situ sampling dates (2016 to 2023).

All preprocessing of S2A and S2B imagery was carried out by the authors and included the following steps: (i) band stacking for each acquisition date; (ii) mosaicking of scenes acquired on the same day; (iii) atmospheric correction using ACOLITE [38], which removes attenuation effects caused by molecular and aerosol scattering and by absorption from water vapor, ozone, oxygen and carbon dioxide [39], (iv) computation of spectral indices (Table 2); and (v) extraction and compilation of reflectance values at the monitoring stations.

#### 2.3.2. PlanetScope Sensor

PS imagery is acquired by small nanosatellites designed and operated by the private company Planet. PS sensors are carried by a constellation of small nanosatellites with a CubeSat 3U form factor (10 × 10 × 30 cm). At present, Planet operates more than 180 Dove nanosatellites that provide daily imagery of Earth′s surface with high spatial resolution (about 3.7 m). Scenes are captured in four spectral bands: B1—Blue (420–530 nm), B2—Green (500–590 nm), B3—Red (610–700 nm), and B4—NIR (780–860 nm). According to Planet Team [61], PS images are delivered orthorectified in the Universal Transverse Mercator projection and geometrically corrected, with about 10 m positional accuracy.

PS scenes were downloaded via the application programming interface (API) available from https://www.planet.com/explorer (accessed on 23 August 2024). We selected cloud-free scenes from the three available sensor generations (Dove Classic, Dove-R, and Super Dove) with acquisition dates coinciding with the water-sampling days between 2016 and 2023. Although PS images are not open access, they can be obtained at no cost through university affiliation by enrolling in the Planet Education and Research program (https://go.planet.com/research, accessed on 1 May 2025).

Preprocessing steps for PS imagery included (i) mosaicking acquisition strips; (ii) calculating spectral indices (Table 3); and (iii) extracting and tabulating data for the water-quality monitoring points.

#### 2.3.3. Normalized PlanetScope Sensor

For the normalized PS dataset, the same PlanetScope sensor data presented in the previous section were used. However, the PS imagery was normalized following the methodology proposed by Dias [36], who developed a procedure to correct radiometric inconsistencies in the PlanetScope constellation′s temporal image series using machine-learning models calibrated with synchronous samples of pseudo-invariant pixels extracted from paired PlanetScope and Sentinel-2 scenes. As a result, the normalized dataset exhibited more stable and comparable temporal series.

#### 2.3.4. Climate Hazards Group Infrared Precipitation with Stations Data

In addition to PS and S2 imagery, we used daily precipitation estimates from the Climate Hazards Group Infrared Precipitation with Stations (CHIRPS) dataset [63]. CHIRPS is a reanalysis product that combines rain-gauge observations with satellite-derived precipitation estimates. It provides global daily data since 1981 at about 0.05° spatial resolution (about 5 km) [64].

Data preparation involved: first, delineating the upstream contributing area for each selected station; second, computing the mean of pixel values within that area; and third, downloading precipitation for the sampling day plus the preceding 14 days (a 15-day window). Accumulated precipitation was then computed in 24 h steps up to 360 h. These accumulated values were added to the database as 15 independent variables for predicting water quality parameters. All image and data preprocessing steps were performed in the R programming language [65].

#### 2.3.5. Acquisition of Reflectance Values

After image preprocessing, we refined and extracted reflectance samples. Surface-water motion can produce direct (sunglint) and diffuse (skyglint) reflection of solar radiation, which markedly affects the spectral response of samples and can overestimate reflectance [66,67]. To reduce this effect in the modeling, pixels with reflectance > 0.6 were assigned NoData values [10].

Reflectance extraction considered two scenarios: (i) For S2 imagery, single-pixel values were extracted. To avoid shoreline interference in water pixels, we selected only stations located on river reaches with a minimum channel width of 30 m (equivalent to three S2 pixels). (ii) For PS imagery, a 3 × 3-pixel window was used, and the mean reflectance over that window was extracted.

### 2.4. Modeling of Water-Quality Parameters Using Machine-Learning Methods

To identify the remote-sensing product most suitable for predicting water quality parameters, modeling was performed using three datasets: (i) S2 imagery; (ii) PS imagery; and (iii) normalized PS imagery, following the methodology proposed by Dias [36].

The structured database was stratified and then randomly split into two subsets: 75% for training and 25% for testing. Stratification ensured sample representativeness according to two main criteria: (i) temporal proportion—training and test sets preserved the proportion of data before and after the dam failure; and (ii) climatic distribution—data were proportionally divided between wet and dry seasons, preserving the same 75/25 proportion within each season, with randomization applied only within each stratum.

Model performance metrics were computed as averages over 100 repetitions for both training and test sets. This procedure is effective for assessing algorithm performance and helps identify problematic samples or outliers in the datasets [10,68,69].

**Figure 2:**
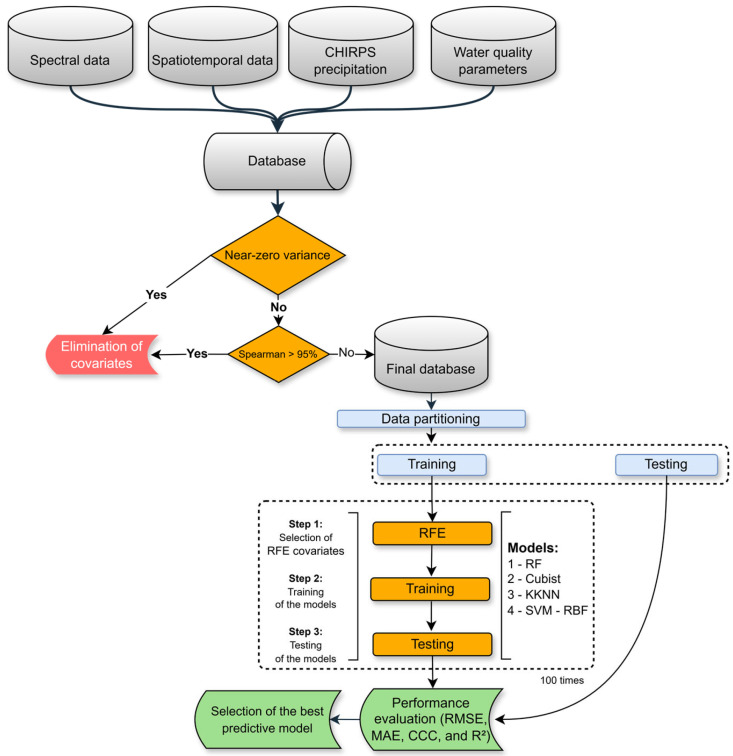
Workflow of the methodology used to predict water-quality parameters from machine-learning algorithms and remote-sensing data. CCC = Lin′s concordance correlation coefficient; CHIRPS = Climate Hazards Group InfraRed Precipitation with Station; R^2^ = coefficient of determination; RMSE = root mean square error; MAE = mean absolute error.

Figure 2 presents a flowchart of the three steps used in the implemented modeling: (i) selecting the optimal set of covariates for each algorithm by removing highly correlated variables and those with lower relevance for training; (ii) training models using the selected variables for each algorithm; and (iii) evaluating model performance on a dataset distinct from that used for training.

#### 2.4.1. Covariate Selection

Covariate selection is a modeling step that aims to identify the smallest subset of original covariates capable of representing the modeled phenomenon/process while minimizing redundancy. It is used to reduce feature-space dimensionality, remove noisy covariates, and increase model parsimony [10,70,71].

First, covariate variance was assessed, and variables with zero or very low variability were removed based on the criteria defined by [72]. This assessment was performed with the nearZeroVar function from the caret package [73,74].

Next, Spearman′s correlation coefficient was computed [75]. For pairs of covariates with correlation ≥ 95%, the variable with the largest absolute correlation with the remaining variables was removed.

Finally, the importance-based removal of covariates was performed through the re-cursive feature elimination (RFE) procedure implemented in the caret package, which dis-cards variables that contribute least to the model according to the algorithm-specific im-portance measures [10,76,77,78,79,80]. The division into training and testing sets was performed prior to the application of the recursive feature elimination (RFE) procedure.

The structured database was stratified and then randomly split into two subsets: 75% for training and 25% for testing. Stratification ensured sample representativeness according to two main criteria: (i) temporal proportion—training and test sets preserved the proportion of data before and after the dam failure; and (ii) climatic distribution—data were proportionally divided between wet and dry seasons, preserving the same 75/25 proportion within each season, with randomization applied only within each stratum.

After applying RFE, the optimal covariate set was obtained for each algorithm and used in the subsequent modeling steps. For modeling water quality parameters, the predictors comprised individual bands, band ratios, spectral indices (Table 2 and Table 3), precipitation data (accumulated from 2 to 15 days prior to sampling), and image-acquisition period (before/after the dam failure and hydrological season).

#### 2.4.2. Selection of Machine-Learning Models

To predict the concentration of water quality parameters, we employed the following algorithms: random forest (RF) [81], support vector machines with a radial basis function kernel (SVM-RBF) [82], kernel k-nearest neighbors (KKNN) [83], and cubist [84]. These algorithms were chosen because they represent distinct families of modeling approaches, providing a comprehensive assessment of the relationships within the data. This set includes: (i) tree-based ensemble methods (RF), (ii) kernel-based methods capable of capturing complex nonlinear relationships (SVM-RBF), (iii) instance-based learning algorithms (KKNN), and (iv) hybrid rule-based models that combine decision trees with linear regression components (Cubist). Such methodological diversity enables the exploration of different response patterns and follows established recommendations in the literature for environmental and limnological modeling, ensuring robustness and comparability across approaches [74,85].

RF and SVM-RBF are widely used to predict water quality parameters [2,86,87]. RF builds an ensemble of *N* regression trees, and the final prediction is the average over all trees. As a tree-based approach, RF is a nonparametric algorithm [81,88].

SVM-RBF allows predictions with a tolerable error controlled by the support vectors and governed by the hyperparameter *C* (cost) [82]. Like RF, SVM-RBF is nonparametric and becomes a nonlinear regression method by using a nonlinear kernel function [89].

KKNN is also kernel-based and identifies the *k* training points closest to a new sample using a distance metric such as Minkowski distance, a general form of Euclidean and Manhattan distances [90]. This nonparametric model assigns distance-weighted contributions so that nearer neighbors receive larger weights, avoiding explicit assumptions about underlying data distributions [91,92,93].

Cubist is a tree-based regression algorithm that combines a decision-tree structure with linear models fit within each terminal region [84]. It builds a set of decision rules to partition the attribute space and then applies local linear regression within each region, yielding a flexible and interpretable representation of relationships in data [94,95,96].

A more detailed description of these algorithms can be found in Kuhn and Johnson [85] and Murphy [74].

During training, each model′s internal hyperparameters were tuned using repeated cross-validation with 10 folds and 10 repetitions, applied to each algorithm′s tuning grid and testing 5 values of each hyperparameter (tuneLength). Hyperparameters are algo-rithm-specific configuration options that influence model behavior and predictive accura-cy. Each learning method relies on its own set of hyperparameters, and in this work we optimized the following: committees and neighbors for Cubist; kmax, distance, and kernel for KKNN; mtry for RF; and sigma and C for the radial-basis SVM-RBF.

The tuning process was carried out automatically through the train function in the caret package [73]. This function performs a structured exploration of the user-defined hyperparameter space. When minimum and maximum values are provided for each parameter, train constructs an evenly spaced grid—typically composed of five candidate values per hyperparameter—covering the specified range. The algorithm is then fitted for every combination in this grid and assessed using the selected resampling strat-egy (e.g., cross-validation). The configuration that maximizes the chosen performance met-ric is retained as the optimal set of hyperparameters.

In this study, hyperparameter selection was guided by the Lin′s Concordance Corre-lation Coefficient (CCC), which served as the optimization criterion. Initial values and search ranges followed the caret developers′ recommendations (see the manual, Chapter “Available Models”: https://topepo.github.io/caret/available-models.html (accessed on 15 January 2025)). Final optimized hyperparameters are shown in Table 4.

The processes of importance-based variable removal (RFE), model training, and performance evaluation were repeated 100 times. This repeated-resampling strategy enables assessing the ability of the algorithms to handle varying training subsets and to produce robust predictive results [97,98]. Model performance metrics for both training and testing sets were then computed as the mean values across the 100 repetitions. This approach enhances the reliability of performance estimates and facilitates the identification of potentially problematic observations or outliers within the datasets [10,68,69].

#### 2.4.3. Model Evaluation

To evaluate model performance, predictions were compared with observations from the water quality monitoring stations in the study area (Table 1) using the following statistical metrics: root-mean-square error (RMSE; Equation (1)), mean absolute error (MAE; Equation (2)), Lin′s concordance correlation coefficient (CCC; Equation (3)), and the coefficient of determination (R^2^; Equation (4)). This set of metrics was chosen to capture complementary facets of performance [99,100,101].

(1) $$RMSE={\left[\frac{1}{n}\sum_{i=1}^{n}{\left(P_{i}-O_{i}\right)}^{2}\right]}^{\frac{1}{2}}$$

(2) $$MAE=\frac{\sum_{i=1}^{n}\left|P_{i}-O_{i}\right|}{n}$$

(3) $$CCC=\frac{2rV_{r}V_{R}}{{\left(\bar{P\;}-\;\bar{O\;}\right)}^{2}+\;V_{r}+V_{R}}$$

(4) $$R^{2}=\frac{\sum_{i=1}^{n}{\left(P_{i}-\bar{O}\right)}^{2}}{\sum_{i=1}^{n}{\left(O_{i}-\bar{O}\right)}^{2}}$$

where $P_{i}$ are model-predicted values; $O_{i}$ are observed values; $\bar{O}$ is the mean of observed values; $\bar{P}$ is the mean of predicted values; $V_{r}$ and $V_{R}$ are the variances of predicted and observed values, respectively; and n is the number of observation pairs.

RMSE squares the difference between predicted and observed values, penalizing large errors more than small ones, and is therefore sensitive to outliers [102]. MAE measures the average magnitude of errors using the absolute difference [103]. CCC quantifies the proximity of the fitted relationship to the 45-degree identity line [101]. R^2^ represents the proportion of variance explained by the model [104]. Because RMSE and MAE share the variable′s units, they facilitate error interpretation. Models with lower RMSE and MAE were considered more accurate [105]. Following Altman [106], CCC and R^2^ can be interpreted as moderate (0.5–0.7), strong (0.7–0.9), and very strong (>0.9).

In addition to these metrics, RMSE (Equation (5)) and MAE (Equation (6)) were also computed for a null model. The null model predicts each parameter using the training-set mean, returning a single average for numeric outcomes. Models performing similarly to or worse than the null model are poorly rated. Model selection for a given parameter should favor cases in which RMSE and MAE are lower than those of the null model, indicating gains from the machine-learning approach [69].

(5) $$NULL\_RMSE={\left[\frac{1}{n}\sum_{i=1}^{n}{\left(\;{\bar{O}}_{mt}-O_{iT}\right)}^{2}\right]}^{\frac{1}{2}}$$

(6) $$NULL\_MAE=\frac{\sum_{i=1}^{n}\left|{\bar{O}}_{mt}-O_{iT}\right|}{n}$$

where ${\bar{O}}_{mt}$ is the mean of the training samples, $O_{iT}$ are the test-set observations, and n is the number of test samples (loop size).

## 3. Results and Discussion

Table 5 presents the descriptive statistics of the water quality parameters used in this study, revealing large gaps between minimum and maximum values—i.e., a wide range in the observed measurements. In addition, the parameter means are generally closer to the minima, indicating right-skewed distributions with long upper tails. Such skewness is common in environmental datasets, where measurements cluster at low to moderate levels but rare extreme events stretch the upper tail [105].

**Figure 3:**
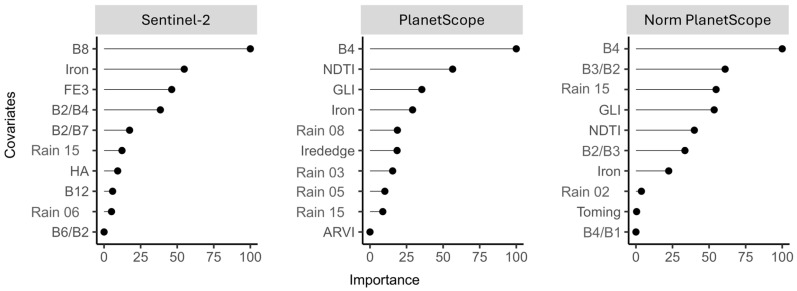
Ranking of the most important covariates for modeling water-quality parameters across the three datasets. Covariates include spectral bands (for example, B3, B4, B8), band ratios (for example, B3/B2, B2/B4), spectral indices (such as GLI, NDTI, ARVI), and environmental variables such as accumulated precipitation prior to the image date (for example, “Rain 15” indicates 15-day rainfall accumulation). ARVI means Atmospherically Resistant Vegetation Index, GLI means Green Leaf Index, and NDTI means Normalized Difference Turbidity Index.

Figure 3 ranks the most important covariates for the models used to predict water quality parameters across the three datasets (S2, PS, and normalized PS). Covariate-selection procedures substantially reduced the number of predictors, from an initial 151 to ten variables per model—an adequate and parsimonious set for modeling. These findings agree with Muñoz-Romero et al. [70] and Stevens et al. [76], who showed that reducing model complexity lowers computational costs and improves robustness and predictive performance.

Except for the NIR bands (B8 for S2 and B4 for PS), indices and band ratios dominate as the most important covariates compared with individual bands. This corroborates Sestini [107] and Lillesand, Kiefer, and Chipman [108], who showed that combining spectral bands via indices and ratios enhances discrimination of subtle spectral differences among targets, whereas individual bands tend to capture only more evident variations—making ratio-based indices more effective for identifying specific spectral features of natural objects or phenomena.

The NIR bands (B8 in Sentinel-2 and B4 in PlanetScope) stand out as the most influential predictors. This result is expected, since NIR reflectance responds strongly to increases in suspended particles, directly influencing the prediction of turbidity, TSS, and other optically active parameters. Even for optically inactive parameters, the NIR band provides indirect information because many chemical components are correlated with sedimentary and hydrodynamic processes, particularly in a post-disaster context where sediment mobilization is intensified.

Spectral indices and band ratios such as GLI, Iron, and NDTI also exhibit high importance. Their superior performance stems from their ability to highlight subtle variations in the water′s spectral response while reducing interference associated with illumination, solar geometry, and atmospheric variability. The Iron index, in particular, consistently appears among the most relevant predictors, reflecting the presence of mineral-rich particulate material that characterizes much of the sediment dynamics in the basin after the disaster. These indices provide a more stable and discriminative spectral signal than individual bands, contributing strongly to the prediction of optically active parameters and, indirectly, to optically inactive ones.

Precipitation-derived variables from the CHIRPS product also appear consistently among the ten most important predictors across all sensors. This behavior reflects the direct relationship between accumulated rainfall, increased surface runoff, sediment transport, and nutrient loading. Precipitation further exerts strong influence on sediment resuspension, especially in lotic environments, altering the optical properties of the water column and, consequently, the spectral response captured by the sensors. In reservoirs, these effects are more attenuated due to longer residence times and lower hydrodynamic energy, which explains the differences observed in model performance between lotic and lentic systems.

In addition to these direct effects on optically active parameters, precipitation also contributes to the prediction of optically inactive parameters through indirect relationships. Rainfall events intensify hydrological processes that mobilize organic matter, nutrients, and sediments, thereby altering optical variables such as turbidity, TSS, and indices sensitive to particulate material. Although these inactive parameters do not exhibit distinct spectral signatures, their variations are associated with these processes, enabling machine-learning models to estimate them indirectly.

Table 6 reports, for MSI/S2 data, the performance metrics for the machine-learning models used to predict water quality parameters in the Paraopeba River Basin.

The results demonstrate the superior robustness of tree-based algorithms, particularly RF and Cubist, when compared with KKNN and SVM-RBF. RF achieved the highest performance for five of the eight parameters, while Cubist ranked within the top two for six parameters. Both models produced the lowest prediction errors (RMSE and MAE) and the highest R^2^ and CCC values, reinforcing the ability of tree-based methods to represent nonlinear relationships and capture multiscale interactions among environmental and hydrological covariates [81]. These findings align with previous studies that emphasize the adaptability of ensemble-based approaches under conditions of high optical and hydrological heterogeneity [104,109,110].

At the parameter level, Turbidity and TSS exhibited the strongest generalization capacity, with CCC values close to 0.82 and 0.72 and R^2^ values ranging from 0.75 to 0.59, accompanied by low RMSE and MAE. Both variables are optically active and strongly governed by suspended-sediment dynamics, which enhances their detectability across multisensor imagery. In contrast, Fe, P, DO, COD, and N showed limited predictive performance (CCC ≈ 0.44–0.31; R^2^ ≈ 0.27–0.15), reflecting their weak or indirect spectral signatures and their sensitivity to short-term hydrological fluctuations. For Chla, all algorithms performed poorly; even the best model (SVM-RBF; CCC = 0.12; R^2^ = 0.05) produced a test-set RMSE higher than the null model.

These results are consistent with the well-known physical–optical limitations of these parameters. DO, Nitrogen, and COD are not optically active and therefore do not exhibit direct spectral signatures detectable by orbital sensors. Their estimation relies on indirect relationships with covariates, which naturally limits model accuracy [67]. In the case of Chla, although characteristic absorption bands exist, its detection in rivers is strongly hindered by low pigment concentrations, high turbidity, and spectral overlap with TSS and CDOM [111,112,113]. These conditions are particularly relevant in the study area, where turbidity remains elevated due to the Brumadinho dam failure, reducing the effective optical depth and weakening the Chla signal.

Overall, there was no evidence of overfitting, as training and test results were concordant. Except for Chla, all parameters showed gains over the null model in RMSE and MAE: RMSE improvements ranged from 47.38% (T) to 6.75% (N); MAE improvements ranged from 61.12% (T) to 7.85% (N). For Chla, no advantage over the null model was observed for RMSE; however, MAE improved by 8.21%.

Table 7 reports, for PS data, the performance metrics for the machine-learning models used to predict water quality parameters in the Paraopeba River Basin.

Table 7 indicates a clear dominance of tree-based models, with Cubist and RF consistently ranking among the top two performers for all eight parameters derived from PS data. For Turbidity, TSS, Fe, and P, CCC values ranged from 0.878 to 0.513 and R^2^ values from 0.796 to 0.337, accompanied by low RMSE and MAE. The close agreement between CCC and R^2^ further reinforces the internal consistency and robustness of the modeling framework [36].

The comparison of RMSE and MAE across training and test sets shows only minor discrepancies, suggesting a low risk of overfitting. As reported in Table 7, RMSE values improved by 52.65 percent to 11.82 percent relative to the null model, while MAE improved by 66.04 percent to 13.82 percent. Similar to the MSI/S2 results, Chla was the only parameter for which the model did not outperform the null model, yielding an RMSE 9.85 percent below the mean and a marginal MAE improvement of 7.28 percent. This reinforces the known difficulty of retrieving Chla from PS imagery in highly turbid and optically complex environments.

Table 8 reports, for normalized PS data, the performance metrics used to evaluate the machine-learning models applied to predicting water quality parameters in the Paraopeba River Basin. For all evaluated parameters, RF and Cubist were among the two best models; only for Fe, P, and Chla did these algorithms perform worse than SVM-RBF and KKNN.

Analyzing model performance by parameter, the models for turbidity, TSS, Fe, P, and DO showed good generalization, with CCC values between 0.918 and 0.553. Corresponding R^2^ values ranged from 0.848 to 0.39. The strong agreement between these two indices is an important indicator of the robustness of the applied methodology.

When analyzing the RMSE and MAE indices, the results show low values and good agreement among the data. Examining the percentage gain of the developed models relative to the NULL RMSE and NULL MAE values, all evaluated parameters demonstrated real improvements, with gains ranging from 59.95% to 13.98% for RMSE. For MAE, the models showed an advantage between 68.77% and 15.04%.

Overall, with the exception of the Chla models derived from S2 and PS datasets, all developed models (Table 6, Table 7 and Table 8) achieved MAE and RMSE values lower than those of the null model, which constitutes the minimum statistical benchmark for acceptable predictive skill [69,85]. This systematic reduction in error metrics indicates that the proposed modeling framework provides a demonstrably superior predictive capability compared with the use of simple mean-based estimates.

The Chla parameter presented the lowest CCC and R^2^ values, indicating significant difficulty for the algorithms to generalize across the three analyzed datasets. From an optical perspective, Chla is often affected by the presence of other Optically Active Components (OACs), such as TSS and colored dissolved organic matter (CDOM) [111,112,113]. In this context, it is noteworthy that the study area was impacted by a mining tailings dam failure, which led to high TSS levels in the datasets used for modeling. This significantly contributed to the poor performance of the machine learning models in predicting the Chla parameter.

Additionally, the characteristics of the predominant soils in the region, classified by Embrapa [114], as Red Latosols, Red-Yellow Latosols, Haplic Cambisols, and Humic Cambisols, directly influence water color and, consequently, its spectral response.

**Figure 4:**
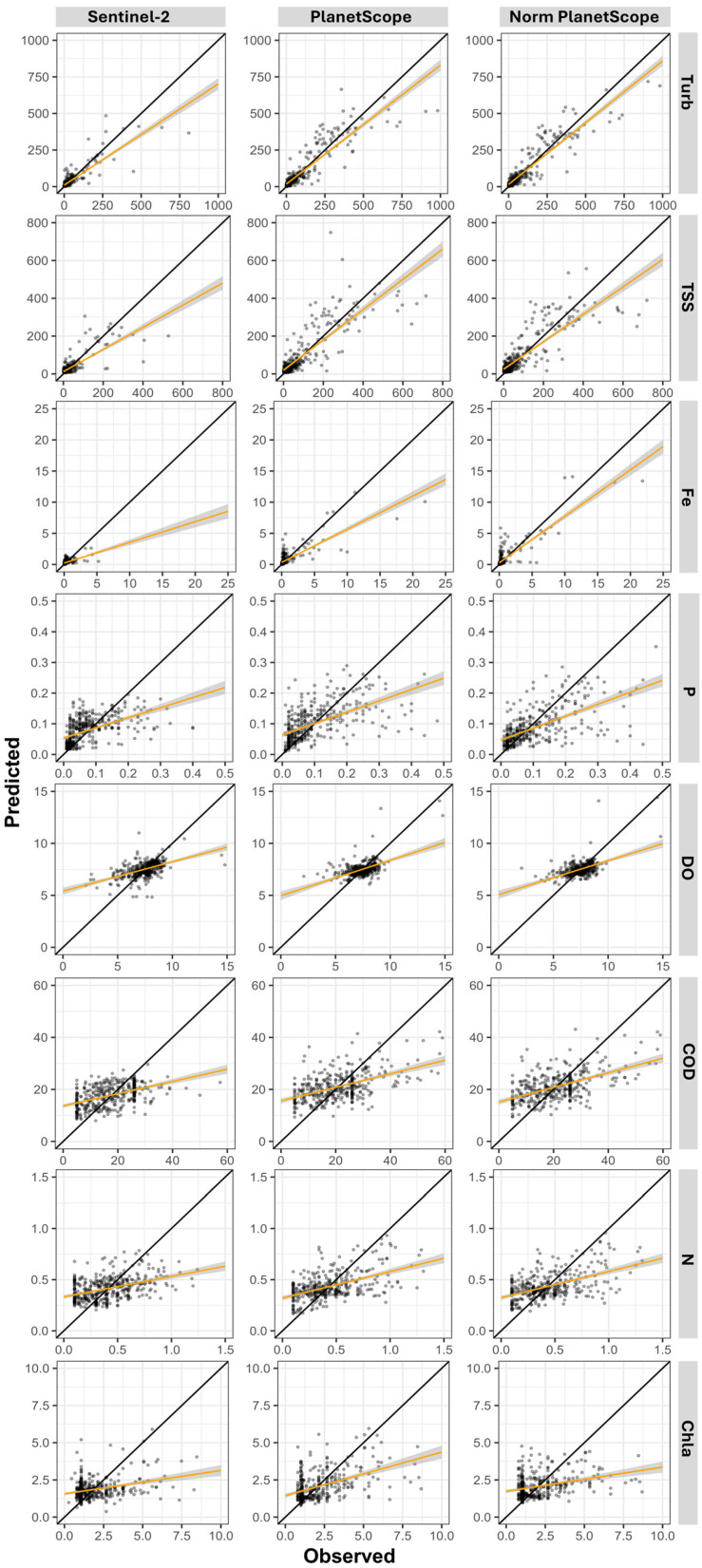
Comparison between predicted and observed reflectance values from the best-performing machine learning models for turbidity (Turb), total suspended solids (TSS), iron (Fe), phosphorus (P), dissolved oxygen (DO), chemical oxygen demand (COD), nitrogen (N), and chlorophyll-a (Chla) across the three analyzed datasets (S2, PS, and normalized PS).

Table 9 presents the performance statistics of the best-performing machine learning models for each evaluated parameter under the three distinct approaches. Figure 4 complements this information by displaying scatter plots of predicted versus observed values along a 1:1 line, allowing a visual assessment of prediction accuracy.

When analyzing Table 9 and Figure 4, the dataset derived from normalized PS images achieved the best results across all evaluated parameters. Models for turbidity, TSS, Fe, P, and DO presented CCC values between 0.92 and 0.55, while R^2^ values ranged from 0.85 to 0.39. The MAE and RMSE values were lower than the thresholds established by the null models. For the parameters COD, N, and Chla, although the results were higher than those obtained with the other two datasets, the CCC values remained below 0.50, varying between 0.45 and 0.26, while R^2^ ranged from 0.30 to 0.11.

These patterns and value magnitudes are consistent with the findings of Gao et al., [115] who predicted non-optically active parameters using S2 data. These results are justified because the predictive capability arises from indirect relationships with optically active constituents and with short-term hydrological dynamics captured by the CHIRPS precipitation covariates. These factors co-vary due to sediment resuspension, nutrient transport, and seasonal changes in streamflow, allowing the models to identify nonlinear environmental patterns.

The results demonstrate the superior performance of the models developed using the PS dataset—both raw and normalized—compared with the S2 data across all analyzed parameters. This advantage arises directly from the characteristics of the PS sensor, such as its high spatial resolution (3.7 m), which enables the detection of finer-scale features, and its daily temporal resolution, which allows for a more accurate characterization of aquatic variability. These findings suggest that PS data offer significant advantages for water quality modeling, especially in complex aquatic systems where spatial and temporal variability is critical.

The MSI/S2 sensor, despite being widely used, has notable limitations in narrow water bodies (< 30 m wide), where its 10 m spatial resolution induces spectral mixing errors. Moreover, its reflectance is highly sensitive to external interferences (riverbed, riparian vegetation, and sediments), as reported by Barbosa et al. [67], Greb et al. [116], and Isidro et al. [117], which reduces its accuracy in more complex aquatic systems.

In summary, when analyzing the characteristics of each sensor across the three evaluated datasets, the results indicate that the models developed using normalized PS data achieved the best performance, surpassing those based on raw PS and MSI/S2 data. This superior performance suggests that normalized PS data are more suitable for predicting water quality parameters, particularly turbidity, TSS, Fe, and P.

**Figure 5:**
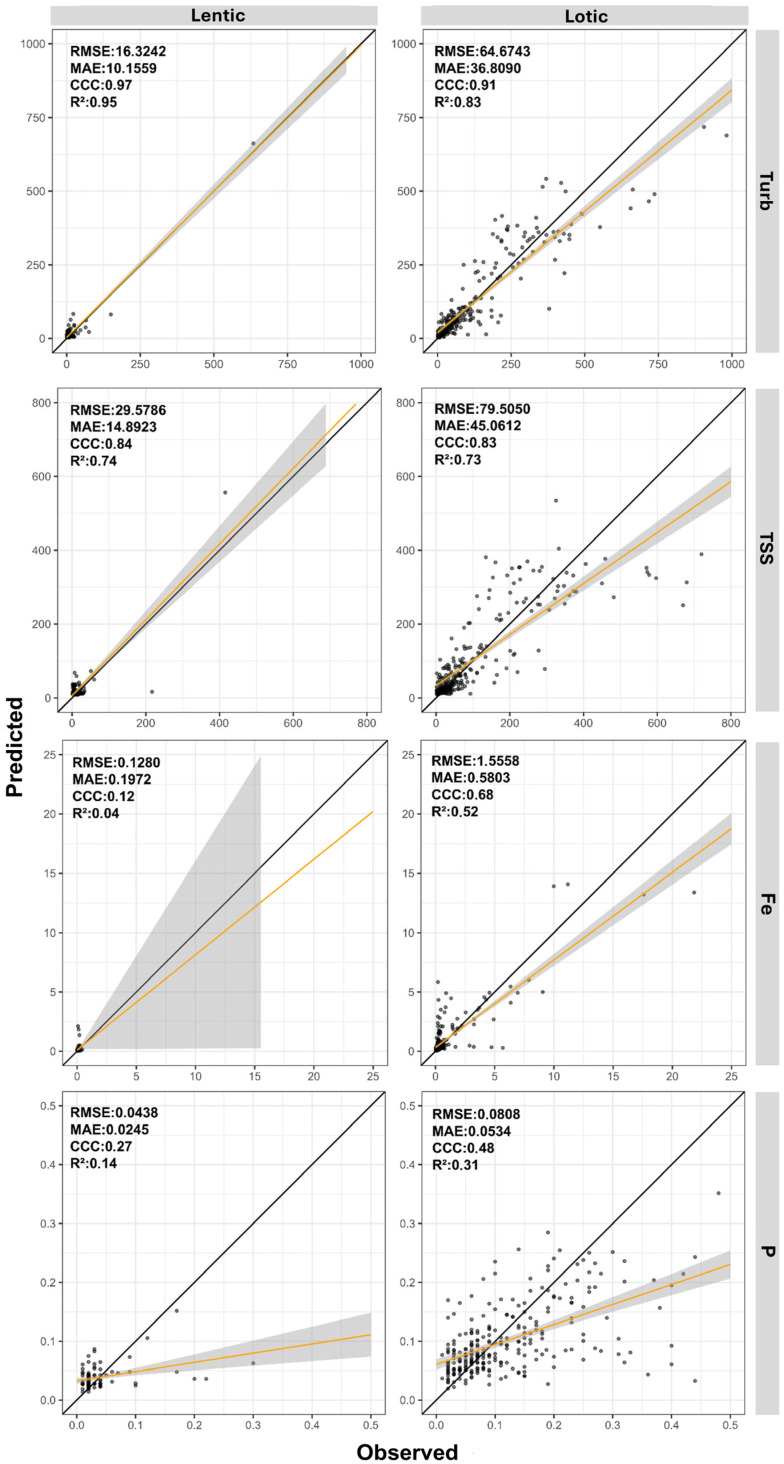
Scatter plots of predicted and observed values for turbidity (Turb), total suspended solids (TSS), iron (Fe), and phosphorus (P) modeled using normalized PlanetScope (PS) data for lentic and lotic environments.

**Figure 6:**
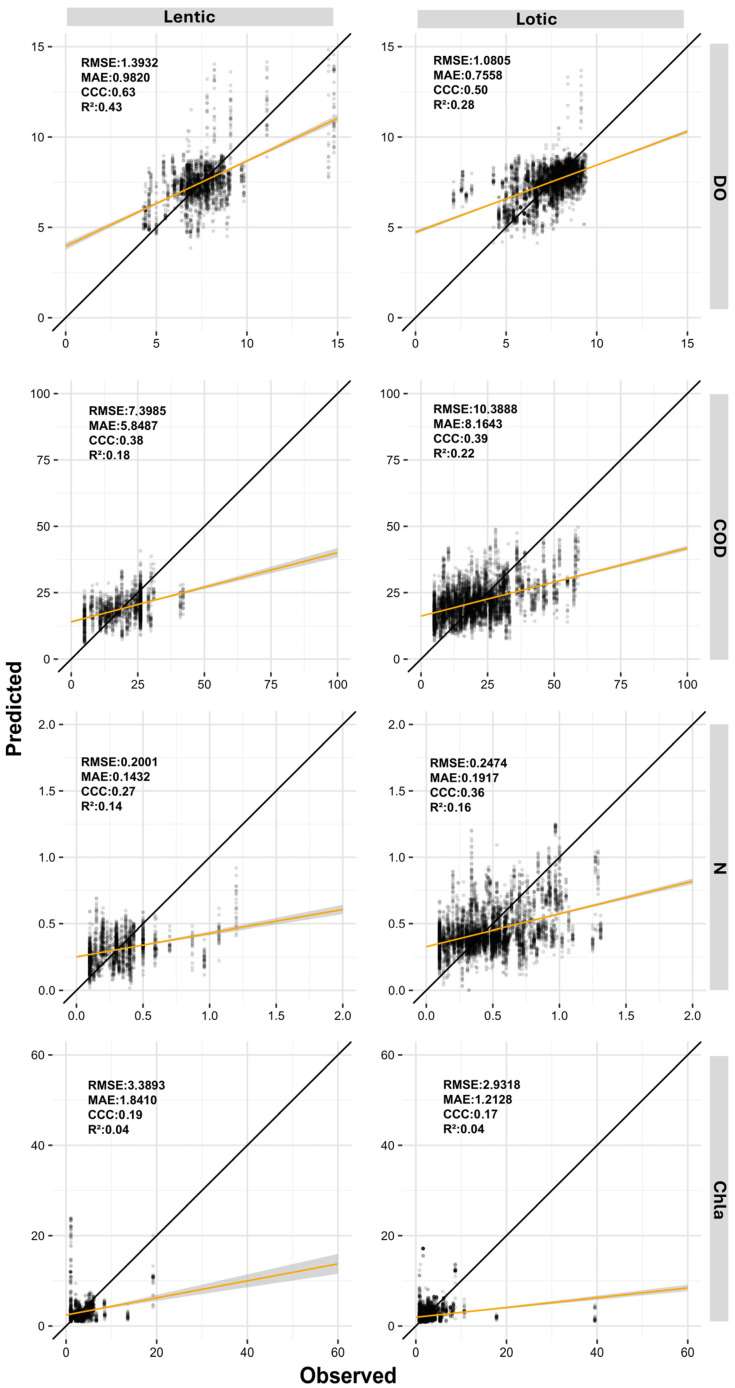
Scatter plots of predicted and observed values for dissolved oxygen (DO), chemical oxygen demand (COD), nitrogen (N), and chlorophyll-a (Chla) modeled using normalized PlanetScope data for lentic and lotic environments. CCC = Lin′s concordance correlation coefficient; MAE = mean absolute error; RMSE = root-mean-square error; R^2^ = coefficient of determination; TSS = total suspended solids; Turb = Turbidity.

Figure 5 and Figure 6 present the predicted and observed values of turbidity, TSS, Fe, P, DO, COD, N, and Chla parameters modeled using normalized PS data for lentic and lotic environments. Overall, due to environmental characteristics, lentic systems exhibited lower dispersion, with values closely grouped. In contrast, lotic environments displayed greater dispersion across all parameters.

Analyzing the statistical indices reveals that turbidity, TSS, COD, and N showed only minor variations in model performance between lentic and lotic environments. Conversely, Fe, P, DO, and Chla exhibited more pronounced differences between the two environments. In lentic systems, turbidity, TSS, and DO achieved the best performances, with CCC values ranging from 0.97 to 0.72 and R^2^ values between 0.95 and 0.65. Meanwhile, Fe, P, COD, N, and Chla had CCC values between 0.43 and 0.12 and R^2^ between 0.28 and 0.04. In lotic environments, turbidity, TSS, and Fe stood out, with CCC values ranging from 0.91 to 0.68 and R^2^ from 0.83 to 0.52. The parameters DO, P, COD, N, and Chla, however, displayed lower CCC and R^2^ values, ranging from 0.48 to 0.19 and 0.31 to 0.06, respectively.

The methodology proposed in this study proved robust for assessing water quality using a historical series of PS images. The comparative analysis between PS data and those from well-established constellation such as S2 demonstrated that PS imagery can provide valuable information despite its lower spectral resolution and the inherent radiometric differences among sensors. Although PS presents certain limitations, it shows strong potential for monitoring water-quality parameters in inland waters, particularly when radiometric normalization is applied. Normalization enhances radiometric consistency across different PS sensor generations (Dove Classic, Dove-R, and Super Dove), reducing calibration discrepancies and improving the temporal comparability of images acquired by different satellites. It also mitigates sensor-specific noise, including variations in gain, offset, and illumination, resulting in more stable and reliable spectral indices that are less susceptible to radiometric distortions.

The proposed methodology not only validates the use of PS data to predict water-quality parameters, but also offers relevant contributions to integrating different data sources to improve predictive accuracy. Based on the evidence presented, Planet′s nanosatellites are a promising tool for environmental monitoring, particularly in contexts that demand continuous, large-scale observations, opening new possibilities for water-resource management and for understanding environmental impacts.

It is important to emphasize that, although PS data stand out for their high spatial resolution and frequent temporal coverage, these images are not free, unlike those provided by the MSI/Sentinel-2 sensor. For this reason, the use of PS imagery requires a careful cost–benefit assessment to determine whether the financial investment is compatible with the monitoring objectives. In the context of this study, the use of PS data was made possible through the Planet Education and Research Program, which justified their inclusion in the analysis. However, in professional applications, the acquisition cost must be weighed against the specific requirements of each monitoring project, considering whether the advantages offered by PS outweigh the free alternative provided by S2.

However, MSI/S2 imagery also presents limitations, such as lower spatial resolution compared with PS, which may hinder the detection of small-scale targets or phenomena. In addition, frequent cloud cover and lower temporal revisit in some regions can limit the applicability of S2 data in studies that require both high spatial and temporal resolution.

## 4. Conclusions

Based on the results, we conclude:

1. The methodology that combines orbital remote-sensing data with machine-learning techniques is suitable for predicting turbidity, TSS, Fe, P, and DO, but shows limitations for N, COD, and Chla.
2. The NIR band was a key covariate in all approaches (B8 for S2, B4 for PS). In addition, spectral indices, band ratios, and CHIRPS precipitation products exhibited strong predictive value for estimating water-quality parameters.
3. To improve model performance with PS data, radiometric normalization of the imagery is essential.
4. Tree-based models—particularly RF and Cubist—were more robust and higher-performing than KKNN and SVM-RBF.

## Figures and Tables

**Table 1:** Identification, geographic coordinates, and operating agencies of the water-quality monitoring stations used in this study.

Station	City	Operator	Longitude *	Latitude *
BP036	Brumadinho	IGAM	591,481.65	7,766,154.53
BP068	Mário Campos	IGAM	582,550.80	7,777,238.56
BP070	Betim	IGAM	577,842.67	7,783,718.41
BP072	Betim	IGAM	571,826.23	7,794,515.43
BP077	Papagaios	IGAM	549,063.79	7,862,981.60
BP078	Curvelo	IGAM	530,977.92	7,880,408.76
BP082	Esmeraldas	IGAM	554,515.30	7,824,940.83
BP083	Papagaios	IGAM	549,278.61	7,858,233.86
BP087	Curvelo	IGAM	528,445.63	7,896,560.69
BP093	Brumadinho	IGAM	587,971.75	7,770,652.15
BP099	Felixlândia	IGAM	521,656.61	7,914,060.68
BPE2	Brumadinho	IGAM	582,099.27	7,773,420.49
BPE6	Felixlândia	IGAM	498,228.23	7,918,958.61
BPE7	Abaeté	IGAM	475,069.83	7,906,716.13
BPE8	Três Marias	IGAM	469,656.80	7,954,599.84
SF011	Biquinhas	IGAM	446,188.74	7,945,567.26
SF054	Três Marias	IGAM	473,291.93	7,988,911.71
TM15	Abaeté	Cemig	478,888.56	7,911,539.85
TM20	Pompéu	Cemig	487,036.46	7,910,337.95
TM25	Pompéu	Cemig	486,013.70	7,917,367.25
TM30	Morada Nova de Minas	Cemig	472,184.16	7,947,127.11
TM35	Morada Nova de Minas	Cemig	461,488.37	7,960,155.51
TM40	Morada Nova de Minas	Cemig	454,887.44	7,955,627.32
TM45	Três Marias	Cemig	473,237.48	7,988,849.00

* Datum: SIRGAS 2000 Universal Transverse Mercator 23S. Cemig = Companhia Energética de Minas Gerais; IGAM = Instituto Mineiro de Gestão das Águas. Source: [35].

**Table 2:** Sentinel-2 spectral indices used as covariates in this study, with their respective equations and bibliographic sources.

Index	Equation	References
Aweish	(B2 + [2.5 × B3]) − (1.5 × [B8 + B12]) − (0.25 × B12)	[40]
WRI	(B3 + B4)/(B8 + B12)	[41]
SCI	(B11 − B8)/(B11 + B8)	[42]
SAVI	([B8 − B4]/[B8 + B4 + 0.5]) × 1.5	[43]
FE_SI	B12/B11	[44]
FE_OX	B11/B8	[44]
FE2	(B12/B8) + (B3/B4)	[45]
FE3	(B12/B8) + (B3/B4)	[45]
NDTI	(B11 − B12)/(B11 + B12)	[46]
NDMI	(B8 − B11)/(B8 + B11)	[47]
NDMI2	(B4 − B8a)/(B4 + B8a)	[48]
Iron	B4/B2	[49]
Clay	B11/B12	[50]
Toming	B3/B4	[51]
NDVI	(B4−B8)/(B4 + B8)	[52]
NDWI	(B3−B8)/(B3 + B8)	[53]
IIA	(4 × B8)/(B3 + [4 × B8])	[54]
IredEdge	(B3 + B4)/2	[55]
GLI	(2 × B3 − B4 − B2)/(2 × B3 + B4 + B2)	[56]
ARVI	(B8−[2 × B4 − B2])/(B8 + [2 × B4] + B2)	[57]
NPQI	(B2 − B3)/(B2 + B3)	[58]
GNDVI	(B5 − B2)/(B5 + B2)	[59]
NDVIVIS	(B2 − B3)/(B2 + B3)	[56]
AWEI	4 × (B3 − B12) − (0.25 × B8a + 2.75 × B11)	[40]
ITBDN	(B3 − B2)/(B3 + B2)	[60]

B corresponds to the Sentinel-2 spectral band used.

**Table 3:** PlanetScope spectral indices used as covariates in this study, with their respective equations and bibliographic sources.

Index	Equation	References
SAVI	((B4 − B3)/(B4 + B3 + 0.5)) × 1.5	[43]
NDTI_VIS	(B3 − B2)/(B3 + B2)	[62]
NDMI2	(B3 − B4)/(B3 + B4)	[47]
Iron	B3/B1	[48]
Toming	B2/B3	[51]
NDVI	(B3 − B4)/(B3 + B4)	[52]
NDWI	(B2 − B4)/(B2 + B4)	[53]
HA	(4 × B4)/(B2 + (4 × B4))	[54]
IredEdge	(B2 + B3)/2	[55]
GLI	(2 × B2 − B3 − B1)/(2 × B2 + B3 + B1)	[56]
ARVI	(B4 − (2 × B3 − B1))/(B4 + (2 × B3) + B1)	[57]
NPQI	(B1 − B2)/(B1 + B2)	[58]
GNDVI	(B4 − B1)/(B4 + B1)	[59]
NDVIVIS	(B1 − B2)/(B1 + B2)	[56]
ITBDN	(B2 − B1)/(B2 + B1)	[60]

B corresponds to the PlanetScope spectral band used.

**Table 4:** Optimized hyperparameters for each developed model.

Parameter	Cubist (Committees/Neighbors)	KKNN (Kmax/Distance/Kernel)	RF (mtry)	SVM-RBF(Sigma/C)
PlanetScope				
Chla	20/5	7/2/optimal	10	0.02427/4
COD	20/5	5/2/optimal	6	18.5751/4
Fe	1/5	5/2/optimal	2	0.50783/2
N	10/5	13/2/optimal	2	0.04940/1
DO	1/9	5/2/optimal	18	16.45009/4
TP	10/9	5/2/optimal	60	0.00942/4
TSS	10/9	7/2/optimal	2	0.03363/2
Turbidity	10/5	13/2/optimal	5	0.008999/2
PlanetScope (normalized)				
Chla	20/5	7/2/optimal	10	0.02427/4
COD	20/5	5/2/optimal	6	18.5853/4
Fe	1/5	5/2/optimal	2	0.50783/2
N	10/5	13/2/optimal	2	0.04940/1
DO	1/9	5/2/optimal	18	16.50009/4
TP	10/9	5/2/optimal	60	0.00942/4
TSS	10/9	7/2/optimal	2	0.03363/2
Turbidity	10/5	13/2/optimal	5	0.008999/2
Sentinel-2				
Chla	1/5	5/2/optimal	2	0.02736/4
COD	1/9	7/2/optimal	6	0.03702/4
Fe	20/5	5/2/optimal	2	0.10574/4
N	20/9	7/2/optimal	18	0.05874/4
DO	20/5	5/2/optimal	5	0.05790/4
TP	20/5	11/2/optimal	11	0.11027/4
TSS	10/9	5/2/optimal	2	0.01212/2
Turbidity	20/9	5/2/optimal	15	0.00732/4

Chla = chlorophyll-a; COD = chemical oxygen demand; DO = dissolved oxygen; Fe = iron; N = nitrogen; P = phosphorus; TSS = total suspended solids.

**Table 5:** Descriptive statistics of water quality parameters for the two datasets used in the modeling.

Sensor	Statistics	Parameters							
		Chla	COD	Fe	P	N	DO	TSS	Turb
MSI/Sentinel-2	Maximum	8.460	57.400	5.241	0.40	1.240	14.800	528.00	809.00
	Minimum	0.270	5.000	0.011	0.010	0.100	2.100	2.00	0.50
	Range	8.190	52.4	5.230	0.390	1.140	12.700	526.00	808.50
	Mean	1.932	17.661	0.317	0.072	0.406	7.481	31.33	34.08
	Median	1.340	17.000	0.174	0.050	0.380	7.700	12.00	11.20
	Std. Deviation	3.718	9.090	0.544	0.080	0.238	1.445	58.20	77.18
PlanetScope	Maximum	8.69	59.000	21.846	0.480	1.310	14.800	716.00	982.00
	Minimum	0.800	5.000	0.023	0.010	0.100	2.100	2.00	0.53
	Range	7.89	54.000	21.823	0.470	1.210	12.700	714.00	981.47
	Mean	2.202	20.777	0.677	0.098	0.431	7.481	78.83	87.93
	Median	1.600	20.000	0.253	0.060	0.380	7.500	29.00	30.45
	Std. Deviation	2.851	10.818	1.937	0.092	0.258	1.300	136.32	144.53

Chla = chlorophyll-a; COD = chemical oxygen demand; DO = dissolved oxygen; Fe = iron; N = nitrogen; P = phosphorus; TSS = total suspended solids; Turb = Turbidity.

**Table 6:** Performance metrics (training/test) for Cubist, KKNN, RF, and SVM-RBF in predicting water quality parameters using Sentinel-2 data.

	Models	Training				Test					
		RMSE	MAE	R^2^	CCC	RMSE	MAE	R^2^	CCC	NULL RMSE	NULL MAE
Turb	Cubist	35.369	14.468	0.787	0.823	42.738	15.210	0.739	0.813	74.451	37.619
	KKNN	36.486	14.856	0.769	0.795	41.701	15.016	0.735	0.799		
	**RF**	**33.953**	**14.322**	**0.785**	**0.824**	**39.176**	**14.625**	**0.750**	**0.824**		
	SVM-RBF	39.347	16.527	0.746	0.761	48.495	17.575	0.656	0.709		
TSS	Cubist	36.185	17.066	0.648	0.703	40.720	17.572	0.560	0.701	57.012	29.762
	**KKNN**	**33.785**	**16.411**	**0.658**	**0.715**	**37.482**	**16.527**	**0.596**	**0.727**		
	RF	34.250	16.800	0.643	0.709	37.878	16.881	0.585	0.719		
	SVM-RBF	34.652	16.899	0.640	0.674	40.932	17.780	0.533	0.630		
Fe	**Cubist**	**0.422**	**0.208**	**0.369**	**0.490**	**0.474**	**0.213**	**0.278**	**0.449**	0.519	0.269
	KKNN	0.438	0.215	0.338	0.466	0.482	0.219	0.242	0.418		
	RF	0.453	0.221	0.299	0.417	0.496	0.225	0.202	0.371		
	SVM-RBF	0.420	0.201	0.341	0.429	0.465	0.201	0.241	0.342		
P	Cubist	0.067	0.042	0.308	0.474	0.071	0.043	0.230	0.420	0.077	0.055
	KKNN	0.068	0.043	0.300	0.471	0.072	0.045	0.210	0.408		
	**RF**	**0.065**	**0.042**	**0.325**	**0.478**	**0.069**	**0.043**	**0.249**	**0.427**		
	SVM-RBF	0.067	0.040	0.299	0.420	0.070	0.040	0.228	0.371		
DO	Cubist	1.320	0.928	0.216	0.380	1.395	0.942	0.175	0.360	1.457	1.006
	KKNN	1.328	0.927	0.215	0.378	1.467	0.985	0.133	0.318		
	**RF**	**1.218**	**0.836**	**0.288**	**0.419**	**1.325**	**0.864**	**0.203**	**0.361**		
	SVM-RBF	1.267	0.857	0.232	0.377	1.382	0.899	0.152	0.316		
COD	Cubist	8.438	6.634	0.192	0.351	8.641	6.746	0.139	0.313	8.978	7.471
	KKNN	8.869	7.067	0.146	0.295	9.124	7.200	0.093	0.252		
	**RF**	**7.969**	**6.340**	**0.247**	**0.381**	**8.026**	**6.366**	**0.212**	**0.369**		
	SVM-RBF	8.566	6.579	0.165	0.317	8.850	6.799	0.106	0.267		
N	Cubist	0.226	0.180	0.172	0.322	0.234	0.184	0.119	0.288	0.237	0.191
	KKNN	0.232	0.185	0.134	0.273	0.241	0.191	0.080	0.227		
	**RF**	**0.213**	**0.171**	**0.220**	**0.352**	**0.221**	**0.176**	**0.159**	**0.313**		
	SVM-RBF	0.231	0.183	0.137	0.280	0.241	0.190	0.082	0.234		
Chla	Cubist	3.406	1.520	0.106	0.156	4.231	1.548	0.047	0.069	3.031	1.364
	KKNN	2.989	1.390	0.149	0.183	3.524	1.397	0.075	0.113		
	RF	3.026	1.468	0.126	0.157	3.501	1.457	0.044	0.077		
	**SVM-RBF**	**2.546**	**1.247**	**0.162**	**0.238**	**3.079**	**1.252**	**0.055**	**0.123**		

CCC = Lin′s concordance correlation coefficient; Chla = chlorophyll-a; COD = chemical oxygen demand; DO = dissolved oxygen; Fe = iron; KKNN = kernel k-nearest neighbors; MAE = mean absolute error; N = nitrogen; NULL MAE = mean absolute error of the null model; NULL RMSE = root-mean-square error of the null model; P = phosphorus; RF = random forest; RMSE = root-mean-square error; R^2^ = coefficient of determination; SVM-RBF = support vector machine with radial basis function; TSS = total suspended solids; Turb = Turbidity. **Note:** Rows in bold and shaded represent the best-performing models for each parameter.

**Table 7:** Performance metrics (training/test) for Cubist, KKNN, RF, and SVM-RBF in predicting water quality parameters using PlanetScope data.

	Models	Training				Test					
		RMSE	MAE	R^2^	CCC	RMSE	MAE	R^2^	CCC	NULL RMSE	NULL MAE
Turb	**Cubist**	**65.317**	**33.266**	**0.825**	**0.878**	**66.664**	**32.608**	**0.796**	**0.878**	140.817	96.021
	KKNN	67.917	34.881	0.803	0.861	76.514	36.639	0.726	0.835		
	RF	65.770	33.961	0.823	0.874	67.549	33.323	0.790	0.873		
	SVM-RBF	66.982	35.895	0.815	0.855	74.055	36.702	0.747	0.830		
TSS	Cubist	76.373	38.527	0.734	0.804	88.274	40.492	0.648	0.768	136.236	86.864
	KKNN	74.968	38.483	0.724	0.798	87.313	41.017	0.627	0.758		
	**RF**	**74.350**	**38.863**	**0.737**	**0.806**	**83.945**	**40.388**	**0.659**	**0.774**		
	SVM-RBF	73.039	38.875	0.737	0.791	84.009	40.907	0.657	0.750		
Fe	**Cubist**	**1.126**	**0.465**	**0.549**	**0.605**	**1.282**	**0.456**	**0.591**	**0.672**	1.866	0.792
	KKNN	1.224	0.446	0.557	0.605	1.367	0.441	0.558	0.671		
	RF	1.408	0.615	0.369	0.421	1.642	0.629	0.315	0.408		
	SVM-RBF	1.172	0.507	0.549	0.573	1.449	0.534	0.505	0.541		
P	Cubist	0.077	0.053	0.342	0.513	0.078	0.053	0.303	0.505	0.091	0.071
	KKNN	0.078	0.053	0.324	0.498	0.079	0.053	0.283	0.487		
	RF	0.075	0.053	0.357	0.494	0.075	0.052	0.327	0.494		
	**SVM-RBF**	**0.075**	**0.049**	**0.362**	**0.510**	**0.076**	**0.048**	**0.337**	**0.513**		
DO	**Cubist**	**1.130**	**0.788**	**0.252**	**0.367**	**1.139**	**0.781**	**0.294**	**0.476**	1.304	0.852
	KKNN	1.159	0.798	0.257	0.385	1.198	0.807	0.270	0.488		
	RF	1.117	0.751	0.249	0.337	1.145	0.750	0.246	0.373		
	SVM-RBF	1.139	0.789	0.231	0.347	1.176	0.805	0.241	0.422		
COD	Cubist	10.324	8.028	0.162	0.303	10.601	8.163	0.127	0.296	10.838	8.487
	KKNN	10.281	7.989	0.183	0.344	10.689	8.191	0.135	0.320		
	**RF**	**9.378**	**7.403**	**0.263**	**0.400**	**9.681**	**7.564**	**0.222**	**0.389**		
	SVM-RBF	9.904	7.699	0.209	0.360	10.397	8.013	0.146	0.322		
N	Cubist	0.226	0.174	0.265	0.417	0.236	0.179	0.202	0.386	0.257	0.202
	KKNN	0.236	0.181	0.223	0.391	0.248	0.189	0.157	0.350		
	**RF**	**0.221**	**0.172**	**0.279**	**0.409**	**0.227**	**0.174**	**0.233**	**0.391**		
	SVM-RBF	0.223	0.171	0.277	0.432	0.235	0.179	0.211	0.394		
Chla	Cubist	2.705	1.448	0.145	0.242	3.010	1.425	0.079	0.179	2.650	1.485
	**KKNN**	**2.758**	**1.429**	**0.167**	**0.267**	**2.911**	**1.377**	**0.124**	**0.257**		
	RF	2.392	1.360	0.159	0.270	2.711	1.361	0.071	0.183		
	SVM-RBF	2.235	1.192	0.217	0.337	2.577	1.212	0.117	0.235		

CCC = Lin′s concordance correlation coefficient; Chla = chlorophyll-a; COD = chemical oxygen demand; DO = dissolved oxygen; Fe = iron; KKNN = kernel k-nearest neighbors; MAE = mean absolute error; N = nitrogen; NULL MAE = mean absolute error of the null model; NULL RMSE = root-mean-square error of the null model; P = phosphorus; RF = random forest; RMSE = root-mean-square error; R^2^ = coefficient of determination; SVM-RBF = support vector machine with radial basis function; TSS = total suspended solids; Turb = Turbidity. **Note:** Rows in bold and shaded represent the best-performing models for each parameter.

**Table 8:** Statistical performance metrics used to evaluate the Cubist, KKNN, RF, and SVM-RBF models for predicting water quality parameters using normalized PlanetScope data.

	Models	Training				Test					
		RMSE	MAE	R^2^	CCC	RMSE	MAE	R^2^	CCC	NULL RMSE	NULL MAE
Turb	**Cubist**	**55.939**	**29.051**	**0.852**	**0.927**	**56.389**	**29.982**	**0.848**	**0.918**	140.817	96.021
	KKNN	66.395	35.054	0.812	0.870	72.223	35.917	0.761	0.856		
	RF	66.144	34.668	0.821	0.873	68.845	34.304	0.784	0.869		
	SVM-RBF	67.443	34.888	0.816	0.854	71.007	34.627	0.771	0.843		
TSS	Cubist	78.835	39.065	0.722	0.793	93.638	38.546	0.614	0.740	136.236	86.864
	KKNN	76.613	39.054	0.713	0.794	90.293	41.962	0.608	0.745		
	**RF**	**69.697**	**36.685**	**0.750**	**0.850**	**70.189**	**37.233**	**0.747**	**0.848**		
	SVM-RBF	75.733	39.432	0.712	0.774	91.637	43.119	0.585	0.701		
Fe	Cubist	1.197	0.489	0.522	0.577	1.345	0.487	0.550	0.653	1.866	0.792
	**KKNN**	**1.211**	**0.478**	**0.528**	**0.589**	**1.084**	**0.424**	**0.656**	**0.764**		
	RF	1.375	0.588	0.409	0.463	1.608	0.609	0.369	0.476		
	SVM-RBF	1.223	0.525	0.506	0.530	1.458	0.537	0.488	0.531		
P	Cubist	0.089	0.053	0.309	0.469	0.090	0.058	0.264	0.456	0.091	0.071
	KKNN	0.078	0.066	0.307	0.472	0.087	0.055	0.258	0.457		
	RF	0.076	0.051	0.335	0.477	0.078	0.054	0.293	0.468		
	**SVM-RBF**	**0.076**	**0.048**	**0.389**	**0.557**	**0.073**	**0.046**	**0.390**	**0.553**		
DO	**Cubist**	**1.150**	**0.798**	**0.238**	**0.353**	**1.017**	**0.704**	**0.392**	**0.557**	1.304	0.852
	KKNN	1.258	0.852	0.175	0.264	1.179	0.780	0.195	0.370		
	RF	1.099	0.739	0.269	0.381	1.114	0.708	0.271	0.402		
	SVM-RBF	1.209	0.815	0.156	0.265	1.259	0.829	0.127	0.257		
COD	Cubist	10.085	7.906	0.195	0.345	9.599	7.470	0.214	0.381	10.838	8.487
	KKNN	10.354	8.172	0.184	0.347	9.392	7.344	0.247	0.422		
	**RF**	**9.252**	**7.289**	**0.280**	**0.414**	**9.041**	**7.140**	**0.302**	**0.445**		
	SVM	9.867	7.827	0.209	0.349	9.234	7.265	0.273	0.401		
N	Cubist	0.226	0.175	0.258	0.404	0.228	0.174	0.228	0.390	0.257	0.202
	KKNN	0.240	0.187	0.193	0.351	0.233	0.180	0.201	0.373		
	**RF**	**0.220**	**0.171**	**0.285**	**0.425**	**0.221**	**0.172**	**0.272**	**0.414**		
	SVM-RBF	0.229	0.178	0.240	0.378	0.232	0.178	0.212	0.383		
Chla	Cubist	2.690	1.461	0.129	0.211	1.923	1.221	0.024	0.150	2.650	1.485
	KKNN	2.621	1.472	0.133	0.221	1.704	1.162	0.070	0.251		
	RF	2.392	1.373	0.150	0.255	1.604	1.169	0.094	0.271		
	**SVM-RBF**	**2.336**	**1.310**	**0.140**	**0.239**	**1.490**	**1.064**	**0.115**	**0.264**		

CCC = Lin′s concordance correlation coefficient; Chla = chlorophyll-a; COD = chemical oxygen demand; DO = dissolved oxygen; Fe = iron; KKNN = kernel k-nearest neighbors; MAE = mean absolute error; N = nitrogen; NULL MAE = mean absolute error of the null model; NULL RMSE = root-mean-square error of the null model; P = phosphorus; RF = random forest; RMSE = root-mean-square error; R^2^ = coefficient of determination; SVM-RBF = support vector machine with radial basis function; TSS = total suspended solids; Turb = Turbidity. **Note:** Rows in bold and shaded represent the best-performing models for each parameter.

**Table 9:** Statistical performance metrics used to evaluate the best-performing models for water quality parameters using Sentinel-2, PlanetScope, and normalized PlanetScope data.

	Data	Models	RMSE	MAE	R^2^	CCC	NULL RMSE	NULL MAE
Turb	S2	RF	39.176	14.626	0.75	0.82	74.451	37.619
	PS	Cubist	66.664	32.609	0.80	0.88	140.817	96.021
	**PS Norm**	**Cubist**	**56.389**	**29.982**	**0.85**	**0.92**	**140.817**	**96.021**
TSS	S2	KKNN	37.482	16.528	0.60	0.73	57.012	29.762
	PS	RF	83.945	40.389	0.66	0.77	136.236	86.864
	**PS Norm**	**RF**	**70.189**	**37.233**	**0.75**	**0.85**	**136.236**	**86.864**
Fe	S2	Cubist	0.474	0.213	0.28	0.45	0.519	0.269
	PS	Cubist	1.282	0.456	0.59	0.67	1.866	0.792
	**PS Norm**	**KKNN**	**1.084**	**0.424**	**0.66**	**0.76**	**1.866**	**0.792**
P	S2	SVM-RBF	0.0690	0.0430	0.25	0.43	0.077	0.055
	PS	SVM-RBF	0.076	0.049	0.34	0.51	0.091	0.071
	**PS Norm**	**SVM-RBF**	**0.073**	**0.046**	**0.39**	**0.55**	**0.091**	**0.071**
DO	S2	RF	1.325	0.864	0.20	0.36	1.457	1.006
	PS	Cubist	1.139	0.781	0.29	0.48	1.304	0.852
	**PS Norm**	**Cubist**	**1.017**	**0.704**	**0.39**	**0.56**	**1.304**	**0.852**
COD	S2	RF	8.026	6.367	0.21	0.37	8.978	7.471
	PS	RF	9.681	7.565	0.22	0.39	10.838	8.487
	**PS Norm**	**RF**	**9.041**	**7.140**	**0.30**	**0.45**	**10.838**	**8.487**
N	S2	RF	0.221	0.177	0.16	0.31	0.237	0.191
	PS	RF	0.227	0.174	0.233	0.391	0.257	0.202
	**PS Norm**	**RF**	**0.221**	**0.172**	**0.27**	**0.41**	**0.257**	**0.202**
Chla	S2	SVM-RBF	3.079	1.253	0.05	0.12	3.031	1.364
	PS	KKNN	2.911	1.378	0.12	0.26	2.650	1.485
	**PS Norm**	**SVM-RBF**	**1.490**	**1.064**	**0.11**	**0.26**	**2.650**	**1.485**

CCC = Lin′s concordance correlation coefficient; Chla = chlorophyll-a; COD = chemical oxygen demand; DO = dissolved oxygen; Fe = iron; KKNN = kernel k-nearest neighbors; MAE = mean absolute error; N = nitrogen; NULL MAE = mean absolute error of the null model; NULL RMSE = root-mean-square error of the null model; P = phosphorus; RF = random forest; RMSE = root-mean-square error; R^2^ = coefficient of determination; SVM-RBF = support vector machine with radial basis function; TSS = total suspended solids; Turb = Turbidity. **Note:** Rows in bold indicate the best-performing model for each parameter.

## Data Availability

The field survey data that supports the findings of this study are available by contacting the corresponding author, RLSD, upon reasonable request.

## References

[1] Ogashawara I., Mishra D.R., Gitelson A.A. (2017). Remote Sensing of Inland Waters: Background and Current State-of-the-Art.

[2] Saberioon M., Brom J., Nedbal V., Souček P., Císař P. (2020). Chlorophyll-a and Total Suspended Solids Retrieval and Mapping Using Sentinel-2A and Machine Learning for Inland Waters. Ecol. Indic..

[3] Barbosa G.R. Introdução Ao Sistema de Informações Geográficas. https://www.kufunda.net/publicdocs/sig-bd-jai.pdf.

[4] Arango J.G., Nairn R.W. (2020). Prediction of Optical and Non-Optical Water Quality Parameters in Oligotrophic and Eutrophic Aquatic Systems Using a Small Unmanned Aerial System. Drones.

[5] Gholizadeh M.H., Melesse A.M., Reddi L. (2016). A Comprehensive Review on Water Quality Parameters Estimation Using Remote Sensing Techniques. Sensors.

[6] Winston R.J., Dorsey J.D., Hunt W.F. (2016). Quantifying Volume Reduction and Peak Flow Mitigation for Three Bioretention Cells in Clay Soils in Northeast Ohio. Sci. Total Environ..

[7] de Aragão R., Cruz M.A.S., Correia E.C.d.O., Machado L.F.M., de Figueiredo E.E. (2017). Impacto Do Uso Do Solo Pelo Aumento Da Densidade Populacional Sobre o Escoamento Numa Área Urbana Do Nordeste Brasileiro via Geotecnologias e Modelagem Hidrológica. Rev. Bras. Geogr. Fís..

[8] Bonansea M., Rodriguez M.C., Pinotti L., Ferrero S. (2015). Using Multi-Temporal Landsat Imagery and Linear Mixed Models for Assessing Water Quality Parameters in Río Tercero Reservoir (Argentina). Remote Sens. Environ..

[9] Cui Y., Yan Z., Wang J., Hao S., Liu Y. (2021). Deep Learning–Based Remote Sensing Estimation of Water Transparency in Shallow Lakes by Combining Landsat 8 and Sentinel 2 Images. Environ. Sci. Pollut. Res..

[10] Dias R.L.S., da Silva D.D., Fernandes-Filho E.I., do Amaral C.H., dos Santos E.P., Marques J.F., Veloso G.V. (2021). Machine Learning Models Applied to TSS Estimation in a Reservoir Using Multispectral Sensor Onboard to RPA. Ecol. Inform..

[11] Sagan V., Peterson K.T., Maimaitijiang M., Sidike P., Sloan J., Greeling B.A., Maalouf S., Adams C. (2020). Monitoring Inland Water Quality Using Remote Sensing: Potential and Limitations of Spectral Indices, Bio-Optical Simulations, Machine Learning, and Cloud Computing. Earth Sci. Rev..

[12] Tian S., Guo H., Xu W., Zhu X., Wang B., Zeng Q., Mai Y., Huang J.J. (2023). Remote Sensing Retrieval of Inland Water Quality Parameters Using Sentinel-2 and Multiple Machine Learning Algorithms. Environ. Sci. Pollut. Res..

[13] Ferdous J., Rahman M.T.U. (2020). Developing an Empirical Model from Landsat Data Series for Monitoring Water Salinity in Coastal Bangladesh. J. Environ. Manag..

[14] Swain R., Sahoo B. (2017). Mapping of Heavy Metal Pollution in River Water at Daily Time-Scale Using Spatio-Temporal Fusion of MODIS-Aqua and Landsat Satellite Imageries. J. Environ. Manag..

[15] Xiong Y., Ran Y., Zhao S., Zhao H., Tian Q. (2020). Remotely Assessing and Monitoring Coastal and Inland Water Quality in China: Progress, Challenges and Outlook. Crit. Rev. Environ. Sci. Technol..

[16] Liu H., Yu T., Hu B., Hou X., Zhang Z., Liu X., Liu J., Wang X., Zhong J., Tan Z. (2021). Uav-Borne Hyperspectral Imaging Remote Sensing System Based on Acousto-Optic Tunable Filter for Water Quality Monitoring. Remote Sens..

[17] El Ouali A., El Hafyani M., Roubil A., Lahrach A., Essahlaoui A., Hamid F.E., Muzirafuti A., Paraforos D.S., Lanza S., Randazzo G. (2021). Modeling and Spatiotemporal Mapping of Water Quality through Remote Sensing Techniques: A Case Study of the Hassan Addakhil Dam. Appl. Sci..

[18] Peterson K.T., Sagan V., Sidike P., Hasenmueller E.A., Sloan J.J., Knouft J.H. (2019). Machine Learning-Based Ensemble Prediction of Water-Quality Variables Using Feature-Level and Decision-Level Fusion with Proximal Remote Sensing. Photogramm. Eng. Remote Sens..

[19] Peterson K.T., Sagan V., Sloan J.J. (2020). Deep Learning-Based Water Quality Estimation and Anomaly Detection Using Landsat-8/Sentinel-2 Virtual Constellation and Cloud Computing. GISci. Remote Sens..

[20] Zhu M., Wang J., Yang X., Zhang Y., Zhang L., Ren H., Wu B., Ye L. (2022). A Review of the Application of Machine Learning in Water Quality Evaluation. Eco Environ. Health.

[21] Gao Y., Gao J., Yin H., Liu C., Xia T., Wang J., Huang Q. (2015). Remote Sensing Estimation of the Total Phosphorus Concentration in a Large Lake Using Band Combinations and Regional Multivariate Statistical Modeling Techniques. J. Environ. Manag..

[22] Sun X., Zhang Y., Shi K., Zhang Y., Li N., Wang W., Huang X., Qin B. (2022). Monitoring Water Quality Using Proximal Remote Sensing Technology. Sci. Total Environ..

[23] Shen Q., Xing X., Yao Y., Wang M., Liu S., Li J., Zhang B. (2021). Estimation of Suspended Matter Concentration in Manwan Reservoir, Lancang River Using Remotely Sensed Small Satellite Constellation for Environment and Disaster Monitoring and Forecasting (HJ-1A/1B), Charge Coupled Device (CCD) Data. Int. J. Remote Sens..

[24] Mathew M.M., Srinivasa Rao N., Mandla V.R. (2017). Development of Regression Equation to Study the Total Nitrogen, Total Phosphorus and Suspended Sediment Using Remote Sensing Data in Gujarat and Maharashtra Coast of India. J. Coast. Conserv..

[25] ESA Guia de Missão Sentinel 2 2023. https://sentinels.copernicus.eu/documents/247904/685211/Sentinel-2_User_Handbook.

[26] NASA Detalhes Da Missão Do Landsat 8 2023. https://science.nasa.gov/mission/landsat-8/.

[27] Nguyen U.N.T., Pham L.T.H., Dang T.D. (2019). An Automatic Water Detection Approach Using Landsat 8 OLI and Google Earth Engine Cloud Computing to Map Lakes and Reservoirs in New Zealand. Environ. Monit. Assess..

[28] Ansper A., Alikas K. (2018). Retrieval of Chlorophyll a from Sentinel-2 MSI Data for the European Union Water Framework Directive Reporting Purposes. Remote Sens..

[29] (2020). Planet Team Planet Surface Reflectance Product v2. https://assets.planet.com/marketing/PDF/Planet_Surface_Reflectance_Technical_White_Paper.pdf.

[30] Crusan J., Galica C. (2019). NASA’s CubeSat Launch Initiative: Enabling Broad Access to Space. Acta Astronaut..

[31] IBGE Instituto Brasileiro de Geografia e Estatística http://www.cidades.ibge.gov.br.

[32] EMBRAPA Sumula Da X Reunião Técnica de Levantamentos de Solos (SNLCS, Série Miscelânia, 1). Serviço Nac. Levant. e Conserv. Solos 1979. https://www.infoteca.cnptia.embrapa.br/infoteca/handle/doc/327212.

[33] Alvares C.A., Stape L., Sentelhas P.C., Gonc L.D.M., Sparovek G. (2014). Koppen’s Climate Classification Map for Brazil. Meteorol. Z..

[34] Gerais G.d.E.d.M. Histórico Do Rompimento Das Barragens Da Vale Na Mina Córrego Do Feijão. https://www.mg.gov.br/pro-brumadinho/pagina/historico-do-rompimento-das-barragens-da-vale-na-mina-corrego-do-feijao.

[35] IGAM Instituto Mineiro de Gestão Das Águas Água Superficial 2021. https://igam.mg.gov.br/w/monitoramento-de-qualidade-das-aguas.

[36] Dias R.L.S., Amorim R.S.S., da Silva D.D., Fernandes-Filho E.I., Veloso G.V., Macedo R.H.F. (2024). Relative Radiometric Normalization for the PlanetScope Nanosatellite Constellation Based on Sentinel-2 Images. Remote Sens..

[37] Müller-Wilm U. (2016). Sentinel-2 MSI—Level-2A Prototype Processor Installation and User Manual.

[38] Vanhellemont Q., Ruddick K. Acolite for Sentinel-2: Aquatic Applications of MSI Imagery. Proceedings of the 2016 ESA Living Planet Symposium.

[39] Gao B., Montes M.J., Davis C.O., Goetz A.F.H. (2009). Atmospheric Correction Algorithms for Hyperspectral Remote Sensing Data of Land and Ocean. Remote Sens. Environ..

[40] Feyisa G.L., Meilby H., Fensholt R., Proud S.R. (2014). Automated Water Extraction Index: A New Technique for Surface Water Mapping Using Landsat Imagery. Remote Sens. Environ..

[41] Mukherjee N.R., Samuel C. (2016). Assessment of the Temporal Variations of Surface Water Bodies in and around Chennai Using Landsat Imagery. Indian J. Sci. Technol..

[42] Li H., Liu Q. Comparison of NDBI and NDVI as Indicators of Surface Urban Heat Island Effect in MODIS Imagery. Proceedings of the International Conference on Earth Observation Data Processing and Analysis.

[43] Huete A.R. (1988). A Soil-Adjusted Vegetation Index (SAVI). Remote Sens. Environ..

[44] Henrich V., Götze E., Jung A., Sandow C., Thürkow D., Gläßer C. Development of an Online Indices Database: Motivation, Concept and Implementation. Proceedings of the 6th EARSeL Imaging Spectroscopy SIG Workshop Innovative Tool for Scientific and Commercial Environment Applications.

[45] Rowan L.C., Mars J.C. (2003). Lithologic Mapping in the Mountain Pass, California Area Using Advanced Spaceborne Thermal Emission and Reflection Radiometer (ASTER) Data. Remote Sens. Environ..

[46] Van Deventer A.P., Ward A.D., Gowda P.H., Lyon J.G. (1997). Using Thematic Mapper Data to Identify Contrasting Soil Plains and Tillage Practices. Photogramm. Eng. Remote Sens..

[47] Zhang K., Thapa B., Ross M., Gann D. (2016). Remote Sensing of Seasonal Changes and Disturbances in Mangrove Forest: A Case Study from South Florida. Ecosphere.

[48] Wilson E.H., Sader S.A. (2002). Detection of Forest Harvest Type Using Multiple Dates of Landsat TM Imagery. Remote Sens. Environ..

[49] Hewson R.D., Cudahy T.J., Huntington J.F. Geologic and Alteration Mapping at Mt Fitton, South Australia, Using ASTER Satellite-Borne Data. Proceedings of the IGARSS 2001. Scanning the Present and Resolving the Future. Proceedings. IEEE 2001 International Geoscience and Remote Sensing Symposium (Cat. No.01CH37217).

[50] Bousbih S., Zribi M., Pelletier C., Gorrab A., Lili-Chabaane Z., Baghdadi N., Ben Aissa N., Mougenot B. (2019). Soil Texture Estimation Using Radar and Optical Data from Sentinel-1 and Sentinel-2. Remote Sens..

[51] Toming K., Kutser T., Laas A., Sepp M., Paavel B., Nõges T. (2016). First Experiences in Mapping Lakewater Quality Parameters with Sentinel-2 MSI Imagery. Remote Sens..

[52] Tucker C.J. (1979). Red and Photographic Infrared Lnear Combinations for Monitoring Vegetation. Remote Sens. Environ..

[53] McFeeters S.K. (1996). The Use of the Normalized Difference Water Index (NDWI) in the Delineation of Open Water Features. Int. J. Remote Sens..

[54] Polidorio A.M., Imai N.N., Tommaselli A.M.G. Índice Indicador de Corpos d’água Para Imagens Multiespectrais. Proceedings of the I Simpósio de Ciências Geodésicas e Tecnologias da Geoinformação (I SIMGEO).

[55] Clevers J., De Jong S.M., Epema G.F., Van Der Meer F.D., Bakker W.H., Skidmore A.K., Scholte K.H. (2002). Derivation of the Red Edge Index Using the MERIS Standard Band Setting. Int. J. Remote Sens..

[56] Costa L., Nunes L., Ampatzidis Y. (2020). A New Visible Band Index (VNDVI) for Estimating NDVI Values on RGB Images Utilizing Genetic Algorithms. Comput. Electron. Agric..

[57] Kaufman Y.J., Tanre D. (1992). Atmospherically Resistant Vegetation Index (ARVI) for EOS-MODIS. IEEE Trans. Geosci. Remote Sens..

[58] Barnes J.D., Balaguer L., Manrique E., Elvira S., Davison A.W. (1992). A Reappraisal of the Use of DMSO for the Extraction and Determination of Chlorophylls a and b in Lichens and Higher Plants. Environ. Exp. Bot..

[59] Gitelson A.A., Kaufman Y.J., Merzlyak M.N. (1996). Use of a Green Channel in Remote Sensing of Global Vegetation from EOS-MODIS. Remote Sens. Environ..

[60] Pizani F.M.C., Maillard P. Um Índice De Turbidez Para Águas Relativamente Claras. Proceedings of the XX Simpósio Brasileiro de Sensoriamento Remoto.

[61] Planet Team (2021). Planet Application Program Interface: In Space for Life on Earth.

[62] Lacaux J.P., Tourre Y.M., Vignolles C., Ndione J.A., Lafaye M. (2007). Classification of Ponds from High-Spatial Resolution Remote Sensing: Application to Rift Valley Fever Epidemics in Senegal. Remote Sens. Environ..

[63] Funk C., Peterson P., Landsfeld M., Pedreros D., Verdin J., Shukla S., Husak G., Rowland J., Harrison L., Hoell A. (2015). The Climate Hazards Infrared Precipitation with Stations—A New Environmental Record for Monitoring Extremes. Sci. Data.

[64] CHIRPS CHIRPS: Rainfall Estimates from Rain Gauge and Satellite Observations|Climate Hazards Center—UC Santa Barbara. https://www.chc.ucsb.edu/data/chirps.

[65] R Core Team (2020). R: A Language and Environment for Statistical Computing, Version 3.3.1.

[66] Carvalho M.L.S., Cabús R.C. (2020). Eficiência Da Luz Solar Refletida e Desempenho de Dispositivos de Sombreamento. Ambient. Construído.

[67] Barbosa C.C.F., Novo E.M.L.M., Martins V.S. (2019). Introdução ao Sensoriamento Remoto de Sistemas Aquáticos.

[68] Neogi S., Dauwels J. (2022). Factored Latent-Dynamic Conditional Random Fields for Single and Multi-Label Sequence Modeling. Pattern Recognit..

[69] Mello D.C.D., Veloso G.V., Lana M.G.D., Mello F.A.D.O., Poppiel R.R., Cabrero D.R.O., Di Raimo L.A.D.L., Schaefer C.E.G.R., Filho E.I.F., Leite E.P. (2022). A New Methodological Framework for Geophysical Sensor Combinations Associated with Machine Learning Algorithms to Understand Soil Attributes. Geosci. Model Dev..

[70] Muñoz-Romero S., Gorostiaga A., Soguero-Ruiz C., Mora-Jiménez I., Rojo-Álvarez J.L. (2020). Informative Variable Identifier: Expanding Interpretability in Feature Selection. Pattern Recognit..

[71] Reunanen J. (2003). Overfitting in Making Comparisons between Variable Selection Methods. J. Mach. Learn. Res..

[72] da Silveira V.A., Veloso G.V., de Paula H.B., dos Santos A.R., Schaefer C.E.G.R., Fernandes-Filho E.I., Francelino M.R. (2022). Modeling and Mapping of Inselberg Habitats for Environmental Conservation in the Atlantic Forest and Caatinga Domains, Brazil. Environ. Adv..

[73] Kuhn M. (2020). Caret: Classification and Regression Training. https://cran.r-project.org/web/packages/caret/index.html.

[74] Murphy K.P. (2013). Machine Learning: A Probabilistic Perspective. Chance Encounters: Probability in Education.

[75] Lee Rodgers J., Nicewander W.A. (1988). Thirteen Ways to Look at the Correlation Coefficient. Am. Stat..

[76] Stevens A., Nocita M., Tóth G., Montanarella L., van Wesemael B. (2013). Prediction of Soil Organic Carbon at the European Scale by Visible and Near InfraRed Reflectance Spectroscopy. PLoS ONE.

[77] Ghosh A., Joshi P.K. (2014). A Comparison of Selected Classification Algorithms for Mappingbamboo Patches in Lower Gangetic Plains Using Very High Resolution WorldView 2 Imagery. Int. J. Appl. Earth Obs. Geoinf..

[78] Meyer H., Reudenbach C., Hengl T., Katurji M., Nauss T. (2018). Improving Performance of Spatio-Temporal Machine Learning Models Using Forward Feature Selection and Target-Oriented Validation. Environ. Model. Softw..

[79] Meyer H., Lehnert L.W., Wang Y., Reudenbach C., Nauss T., Bendix J. (2017). From Local Spectral Measurements to Maps of Vegetation Cover and Biomass on the Qinghai-Tibet-Plateau: Do We Need Hyperspectral Information?. Int. J. Appl. Earth Obs. Geoinf..

[80] Gomes L.C., Faria R.M., de Souza E., Veloso G.V., Schaefer C.E.G.R., Fernandes Filho E.I. (2019). Modelling and Mapping Soil Organic Carbon Stocks in Brazil. Geoderma.

[81] Breiman L. (2001). Random Forests. Mach. Learn..

[82] Cortes C., Vapnik V. (1995). Support-Vector Networks. Mach. Learn..

[83] Zhang M.L., Zhou Z.H. (2007). ML-KNN: A Lazy Learning Approach to Multi-Label Learning. Pattern Recognit..

[84] Adams A., Sterling L. AI ’92. Proceedings of the 5th Australian Joint Conference on Artificial Intelligence.

[85] Kuhn M., Johnson K. (2013). Applied Predictive Modeling.

[86] Tiyasha T., Tung T.M., Bhagat S.K., Tan M.L., Jawad A.H., Mohtar W.H.M.W., Yaseen Z.M. (2021). Functionalization of Remote Sensing and On-Site Data for Simulating Surface Water Dissolved Oxygen: Development of Hybrid Tree-Based Artificial Intelligence Models. Mar. Pollut. Bull..

[87] Du C., Wang Q., Li Y., Lyu H., Zhu L., Zheng Z., Wen S., Liu G., Guo Y. (2018). Estimation of Total Phosphorus Concentration Using a Water Classification Method in Inland Water. Int. J. Appl. Earth Obs. Geoinf..

[88] Ferreira R.G., da Silva D.D., Elesbon A.A.A., Fernandes-Filho E.I., Veloso G.V., Fraga M.d.S., Ferreira L.B. (2021). Machine Learning Models for Streamflow Regionalization in a Tropical Watershed. J. Environ. Manag..

[89] Mountrakis G., Im J., Ogole C. (2011). Support Vector Machines in Remote Sensing: A Review. ISPRS J. Photogramm. Remote Sens..

[90] Cover T., Hart P. (1967). Nearest Neighbor Pattern Classification. IEEE Trans. Inf. Theory.

[91] Abdulrahman S.A., Khalifa W., Roushdy M., Salem A.-B.M. (2020). Comparative Study for 8 Computational Intelligence Algorithms for Human Identification. Comput. Sci. Rev..

[92] Dudani S.A. (1976). The Distance-Weighted k-Nearest-Neighbor Rule. IEEE Trans. Syst. Man. Cybern..

[93] Shepard D. A Two-Dimensional Interpolation Function for Irregularly-Spaced Data. Proceedings of the 1968 23rd ACM National Conference.

[94] Noi P.T., Degener J., Kappas M. (2017). Comparison of Multiple Linear Regression, Cubist Regression, and Random Forest Algorithms to Estimate Daily Air Surface Temperature from Dynamic Combinations of MODIS LST Data. Remote Sens..

[95] Houborg R., McCabe M.F. (2018). A Hybrid Training Approach for Leaf Area Index Estimation via Cubist and Random Forests Machine-Learning. ISPRS J. Photogramm. Remote Sens..

[96] Hafeez S., Wong M.S., Ho H.C., Nazeer M., Nichol J., Abbas S., Tang D., Lee K.H., Pun L. (2019). Comparison of Machine Learning Algorithms for Retrieval of Water Quality Indicators in Case-II Waters: A Case Study of Hong Kong. Remote Sens..

[97] de Mello D.C., Francelino M.R., Moquedace C.M., Baldi C.G.O., Silva L.V., Siqueira R.G., Veloso G.V., Fernandes-Filho E.I., Thomazini A., Demattê J.A.M. (2025). Global Warming May Turn Ice-Free Areas of Maritime and Peninsular Antarctica into Potential Soil Organic Carbon Sinks. Commun. Earth Environ..

[98] Kennedy J.B., Neville A.M. (1986). Basic Statistical Methods for Engineers and Scientists.

[99] Arlot S., Celisse A. (2010). A Survey of Cross-Validation Procedures for Model Selection. Stat. Surv..

[100] Lin L.I.-K. (1989). A Concordance Correlation Coefficient to Evaluate Reproducibility. Biometrics.

[101] Chai T., Draxler R.R. (2014). Root Mean Square Error (RMSE) or Mean Absolute Error (MAE)?—Arguments against Avoiding RMSE in the Literature. Geosci. Model Dev..

[102] Willmott C.J., Matsuura K. (2005). Advantages of the Mean Absolute Error (MAE) over the Root Mean Square Error (RMSE) in Assessing Average Model Performance. Clim. Res..

[103] Morse-McNabb E.M., Hasan M.F., Karunaratne S. (2023). A Multi-Variable Sentinel-2 Random Forest Machine Learning Model Approach to Predicting Perennial Ryegrass Biomass in Commercial Dairy Farms in Southeast Australia. Remote Sens..

[104] Bruce A., Bruce P. (2019). Estatística Prática Para Cientistas de Dados.

[105] Wilks D.S. (2011). Statistical Methods in the Atmospheric Sciences.

[106] Altman D.G. (1990). Practical Statistics for Medical Research.

[107] Sestini M.F. (1999). Variáveis Geomorfológicas no Estudo de Deslizamentos em Caraguatatuba-SP Utilizando Imagens TM-Landsat e SIG.

[108] Lillesand T., Kiefer R.W., Chipman J. (2015). Remote Sensing and Image Interpretation.

[109] Tyralis H., Papacharalampous G., Langousis A. (2019). A Brief Review of Random Forests for Water Scientists and Practitioners and Their Recent History in Water Resources. Water.

[110] Csillik O., Kumar P., Mascaro J., O’Shea T., Asner G.P. (2019). Monitoring Tropical Forest Carbon Stocks and Emissions Using Planet Satellite Data. Sci. Rep..

[111] Bertone E., Ajmar A., Giulio F., Dunn R.J.K., Nicholas J., Doriean C., Bennett W.W., Purandare J. (2024). Satellite-Based Estimation of Total Suspended Solids and Chlorophyll-a Concentrations for the Gold Coast Broadwater, Australia. Mar. Pollut. Bull..

[112] Dall’Olmo G., Gitelson A.A., Rundquist D.C., Leavitt B., Barrow T., Holz J.C. (2005). Assessing the Potential of SeaWiFS and MODIS for Estimating Chlorophyll Concentration in Turbid Productive Waters Using Red and Near-Infrared Bands. Remote Sens. Environ..

[113] Kutser T., Pierson D.C., Kallio K.Y., Reinart A., Sobek S. (2005). Mapping Lake CDOM by Satellite Remote Sensing. Remote Sens. Environ..

[114] (2006). EMBRAPA Sistema Brasileiro de Classificação de Solos.

[115] Gao L., Shangguan Y., Sun Z., Shen Q., Shi Z. (2024). Estimation of Non-Optically Active Water Quality Parameters in Zhejiang Province Based on Machine Learning. Remote Sens..

[116] Greb S., Dekker A.G., Binding C., Bernard S., Brockmann C., DiGiacomo P., Griffith D., Groom S., Hestir E., Hunter P. (2018). Earth Observations in Support of Global Water Quality Monitoring.

[117] Isidro C.M., McIntyre N., Lechner A.M., Callow I. (2018). Quantifying Suspended Solids in Small Rivers Using Satellite Data. Sci. Total Environ..

